# Functional Characterization of Lysophosphatidylcholine: Acyl-CoA Acyltransferase Genes From Sunflower (*Helianthus annuus* L.)

**DOI:** 10.3389/fpls.2020.00403

**Published:** 2020-04-15

**Authors:** Ana Mapelli-Brahm, Rosario Sánchez, Xue Pan, Antonio J. Moreno-Pérez, Rafael Garcés, Enrique Martínez-Force, Randall J. Weselake, Joaquín J. Salas, Mónica Venegas-Calerón

**Affiliations:** ^1^Instituto de la Grasa (CSIC), Campus Universitario Pablo de Olavide, Seville, Spain; ^2^Department of Agricultural, Food and Nutritional Science, 410 Agriculture/Forestry Centre, University of Alberta, Edmonton, AB, Canada; ^3^Departamento de Genética, Facultad de Biología, Universidad de Sevilla, Seville, Spain

**Keywords:** acyl editing, desaturation, *Helianthus annuus*, linoleate, LPCAT, lysophosphatidylcholine acyltransferase, oil synthesis, sunflower

## Abstract

Lysophosphatidylcholine acyltransferase (LPCAT, EC 2.3.1.23) is an evolutionarily conserved key enzyme in the Lands cycle that catalyzes acylation of lysophosphatidylcholine (LPC) to produce phosphatidylcholine (PC), the main phospholipid in cellular membranes. In this study, three *LPCAT* genes from sunflower were identified and the corresponding proteins characterized. These *HaLPCAT* genes encoded functionally active enzymes that were able to complement a deficient yeast mutant. Moreover, enzymatic assays were carried out using microsomal preparations of the yeast cells. When acyl specificities were measured in the forward reaction, these enzymes exhibited a substrate preference for unsaturated acyl-CoAs, especially for linolenoyl-CoA, while in the reverse reaction, linoleoyl or linolenoyl acyl groups were transferred from PC to acyl-CoA to a similar extent. Expression levels of *LPCAT* genes were studied revealing distinct tissue-specific expression patterns. In summary, this study suggests that the combined forward and reverse reactions catalyzed by sunflower LPCATs facilitate acyl-exchange between the *sn*-2 position of PC and the acyl-CoA pool. Sunflower LPCATs displayed different characteristics, which could point to different functionalities, favoring the enrichment of seed triacylglycerols (TAGs) with polyunsaturated fatty acid (PUFA).

## Introduction

Oleaginous seeds accumulate TAGs as reserve material which is used to feed the embryo during germination (Lu et al., 2011; Chapman and Ohlrogge, 2012). Fatty acids are synthesized *de novo* via the action of acetyl-CoA carboxylase and the fatty acid synthase complex in the plastids (Ohlrogge and Browse, 1995). The fatty acids, which are mainly comprised of palmitate, stearate, and oleate, are exported to the outside of the plastid where they are esterified to acyl-CoAs via the catalytic action of long chain acyl-CoA synthetases (Shockey et al., 2002; Chapman and Ohlrogge, 2012). Acyl-CoAs are central metabolites in plant lipid metabolism, and they are the main donor of acyl moieties for glycerolipid synthesis.

The first pathway depicted for glycerolipid biosynthesis involves the successive acylation of G3P with acyl moieties coming from the acyl-CoA pool in the endoplasmic reticulum (ER) (Kennedy, 1961). The first and second acylation reactions of G3P are catalyzed by GPAT (Zheng et al., 2003) and LPAAT (Kim and Huang, 2004), respectively. ER PAP (Eastmond et al., 2010) then catalyzes the liberation of the phosphate group from phosphatidate to produce DAG. The third and last reaction of acylation can be catalyzed by ER-bound DGAT (Hobbs et al., 1999; Lu et al., 2003) or PDAT (Dahlqvist et al., 2000) which produce the TAGs that are accumulated in oil bodies. In the case of sunflower, a previous work pointed for a higher contribution of DGAT to TAG synthesis in this species (Banaś et al., 2013). Among the intermediates of this pathway, phosphatidate is the precursor for some important membrane glycerolipids including PS, PE, phosphatidylglycerol, and PI (Rontein et al., 2001; Justin et al., 2002; Yamaoka et al., 2011) whereas DAG is precursor of PC (Goode and Dewey, 1999), which is the main component of extraplastidial membranes. A remarkable fact within plant lipid metabolism is the synthesis and routing of PUFAs from the lipid species in which they are synthesized (i.e., PC) to the reserve TAGs. These PUFAs, which are mainly linoleate and linolenate, are synthesized by the catalytic action of FAD 2 and FAD3 (Sperling et al., 1993; Shah et al., 1997; Shanklin and Cahoon, 1998) by successive desaturation of oleate in the *sn*-2 and *sn-1* positions of PC. The pathways involved in the channeling and transport of those fatty acid to TAGs from PC are one of the keys for the accumulation of fatty acids of interest in engineered oil crops, including PUFAs and other PC-modified fatty acids (e.g., ricinoleic acid) (Thelen and Ohlrogge, 2002). LPCAT (EC 2.3.1.23) activity is important in this context. LPCAT enzymes catalyze the reacylation of LPC using acyl moieties coming from the acyl-CoA pool (Stymne and Stobart, 1987; Bates and Browse, 2012). Together with phospholipases, these enzymes operate within the Lands cycle (Lands, 1965; Wang et al., 2012) to help maintain an active and continuous turnover of fatty acids in cellular membranes. Moreover, LPCATs are involved in the primary incorporation of the *de novo*-synthesized fatty acids into plant glycerolipids through acyl-editing processes, which eventually support higher metabolic fluxes into glycerolipids than the Kennedy pathway (Bates et al., 2007). A later work on soybean developing embryos showed that TAG synthesis in oil accumulating tissues involves different pools of DAG and PC. Thus, it was experimental evidence of a large bulk PC pool created by acyl edition through the action of LPCATs coexisting with a smaller active PC pool coming from *de novo* DAG synthesis. Most TAGs synthesized in that tissue come from acylation of DAG associated to the active PC pool (Bates et al., 2009; Bates and Browse, 2012). The role of LPCAT enzymes in the transfer of PUFAs from PC to TAG was reinforced by results published by Bates et al. (2012). In that work, an *Arabidopsis thaliana* (hereafter Arabidopsis) double mutant *Atlpcat1/Atlpcat2* displayed lower levels of PUFAs than the WT in the seed oil, with increased proportions of oleic acid and VLCFAs. This phenotype was enhanced when the *ROD1* gene encoding PDCT was also knocked out. The triple mutant *Atlpcat1*/*Atlpcat2*/*Atrod1* displayed a more severe reduction in the content of PUFAs (Bates et al., 2012). LPCAT can also catalyze the formation of acyl-CoA and LPC from PC and free CoA under certain conditions, in what is known as its reverse reaction (Stymne and Stobart, 1984), which has been shown to display substrate specificities different from the forward reaction (Lager et al., 2013). LPCAT homologs have been investigated in *Brassica napus* and *Camelina sativa* (Zheng et al., 2012; Kliñska et al., 2019). Another important role proposed for LPCAT was supplying LPC for the synthesis of plastidial galactolipids (Mongrand et al., 1997, 2000), which is an important aspect of lipid trafficking between the ER and chloroplasts. This mechanism would allow the import of glycerolipids enriched in linolenic acid synthesized in the ER within the chloroplasts, interconnecting the so-called prokaryotic glycerolipid pathway, specific of this organelle and the eukaryotic one, proper of the ER. Recent characterization of the acyl fluxes in the Arabidopsis mutant *act1/Atlpcat1/Atlpcat2*, which combines the absence of LPCAT activity with the blockage of the prokaryotic pathway, indicates that LPCAT activity is not involved in the transfer of LPC from the ER to the chloroplast (Karki et al., 2019). Indeed, the lipid transference between those organelles took place by direct turnover of a certain active pool of PC into monogalactosyl diacylglycerol (MGDG). That pool was different from the bulk PC pool participating in the acyl editing pathway involving LPCATs.

Sunflower (*Helianthus annuus* L.) is an oil crop accumulating large amounts of oil rich in oleic and linoleic acids in their seeds. The synthesis of TAGs in sunflower has been extensively investigated at both molecular and biochemical levels (Salas et al., 2014; Venegas-Calerón et al., 2015), although little is known about the pathways of acyl editing and routing of linoleoyl moieties of PC to reserve TAGs. There are some early studies on sunflower LPCAT. In the first one, Sperling et al. (1993) depicted the incorporation and desaturation of radiolabeled 18:1-CoA to PC in sunflower microsomes. In a later work, Fraser and Stobart (2000) partialy purified and characterized LPCAT from seed microsomes of this species.

In the present work, three different sunflower *LPCAT* genes expressed at high levels in developing cotyledons were cloned and functionally characterized. Their implication and contribution to the acyl editing of sunflower glycerolipids and the synthesis of sunflower TAGs are discussed.

## Materials and Methods

### Biological Materials and Growth Conditions

Sunflower (*H. annuus* L.) plants of CAS-6 were cultivated in growth chambers as described elsewhere (Troncoso-Ponce et al., 2018).

All Arabidopsis plants used in this study were Columbia ecotype. The double mutants *lpcat1 lpcat2-2* (SALK-123480 and Sail_1213G01) previously described by Wang et al. (2012) and *lpcat1 lpcat2* (SALK-123480 and Sail_357_H01) of Bates et al. (2012) were used in this work. Arabidopsis transgenic seeds were germinated on vertically positioned agar-solidified Murashige and Skoog media (Murashige and Skoog, 1962) containing 50 μg ml^−1^ kanamycin. Seeds sown on plates were stratified at 4°C for 3 days. Later, the seedlings were transferred to compost and grown in a chamber under a 16/8 h light/dark cycle photoperiod.

The host strain used for the production of the recombinant LPCAT enzymes in yeast was the *Saccharomyces cerevisiae* haploid knock-out mutant ALE1 (or *slc4*, or YOR175c, BY4741; Mata; his3Δ1; leu2Δ0; met15Δ0; ura3Δ0; YOR175c:kanMX4^1^) obtained from the EUROSCARF collection (Acc. No. Y02431). The *S. cerevisiae slc1*Δ *slc4*Δ double mutant, FBY4137 (his3Δ1 leu2Δ0 ura3Δ0 slc1:kanMX4 yor175c:LEU2), was used to test LPAAT activity (Benghezal et al., 2007). To avoid the lethality of the simultaneous deletion of *SLC1* and SLC4, this double mutant harbor the pGREG546:*Sc*SLC1 construct (Jansen et al., 2005), that allow the expression of *Sc*SLC1 under the GAL1 promoter.

### Gene Cloning and Sequence Analysis

Putative genes encoding LPCAT from *H. annuus* were searched on the basis of the amino acid sequences for the homologous proteins from *A. thaliana* (*At*LPCAT1: NP_172724; At1g12640 and *At*LPCAT2: NP_176493; At1g63050). These sequences were used as queries against Expressed Sequence Tags database from the NCBI^2^. Two sunflower *LPCAT* homologous genes were identified (denoted as *HaLPCAT1* and *HaLPCAT2*). Using these sequences, *H. annuus* sequences were searched in the French National Institute for Agricultural Research (INRA) database^3^, allowing to deduce the sequence of the coding region of a third isoform, *HaLPCAT3*.

In order to obtain the complete cDNA sequence of each gene, a pair of specific oligonucleotides for each isoform was designed (Supplementary Table S1). The coding regions were amplified by PCR and directionally cloned into the yeast expression vectors pYES2 (Thermo Fisher Scientific, United States) and p423GPD (Mumberg et al., 1995). To assess *Ha*LPCAT functionality by heterologous expression in the Arabidopsis double *lpcat1*/*lpcat2* mutant. Sunflower cDNAs encoding LPCATs were inserted into the binary expression vectors pBIN19:35S (Sayanova et al., 1997).

### Sequence Analysis and Phylogenetic Tree Constructions

The deduced amino acid sequences of *Ha*LPCAT proteins were identified by alignment to homologous sequences from other plant species found in the NCBI database^2^ using the BLAST 2.0 program (Altschul et al., 1990). The alignment was performed using ClustalX v2.0.10 program (Larkin et al., 2007) under the default settings and further modifications on the alignments were performed with Bioedit Sequence Alignment Editor Program (Hall, 1999). The Conserved Domain Database (CDD; Marchler-Bauer et al., 2017) which is part of the search system of the BLAST 2.0 program of the NCBI server, allowed to identify the different proteins as belonging to a known superfamily.

The phylogenetic analysis was performed using the neighbor-joining method from MEGA 6 (Tamura et al., 2013).

Transmembrane domains were predicted using the bioinformatics program TopPred II (von Heijne, 1992; Claros and Heijne, 1994), available on the Bioinformatics Resource Portal ExPASy^4^.

### Analysis of Tissue-Specific LPCAT Gene Expression by qRT-PCR

For the quantitative expression studies seeds from 12 to 28 DAF, cotyledons, roots, stems, and leaves were collected. RNA extraction and cDNA synthesis were performed as described by Aznar-Moreno et al. (2018). The cDNA samples were subjected to real time quantitative PCR (qRT-PCR) with specific pair of primers for the genes coding for *Ha*LPCAT1, *Ha*LPCAT2, and *Ha*LPCAT3 (Supplementary Table S1) as described by González-Mellado et al. (2019). The method of Livak and Schmittgen (2001) was used to calculate the relative expression levels of the studied genes. The sunflower *ACTIN* gene was used as calibrator gene and was amplified with the primers shown in Supplementary Table S1.

### Yeast Complementation Assays

A lyso-PAF sensitivity test (Chen et al., 2007) was carried out with the *S. cerevisiae* mutant strain Y02431 (*slc1*Δ) transformed with the recombinant plasmid pYES2 containing the sunflower *LPCAT* genes. S288C yeast was used as positive control. Cultures were grown overnight in SC-Ura containing 2% (w/v) glucose at 30°C. Cultures were then diluted to DO_600_ of 0.4 in SC-Ura containing 2% (w/v) galactose and 1% (w/v) raffinose for expression induction. After 24 h of induction, cultures were serial diluted 1:10 to DO_600_ = 2 × 10^−4^. Volumes of 10 μl of each dilution were plated onto SC-Ura plates containing 2% (w/v) galactose, 1% (w/v) raffinose, and 0, 5, or 10 μg/ml lyso-PAF. The plates were incubated at 30°C for 72 h.

An assay of counter-selection in the presence of 5-FOA was carried out with the double mutant strain FBY4137 (harboring the pGREG546:*Sc*SLC1 construct) transformed with the *HaLPCAT* genes-containing p423GPD plasmid. Cultures were grown overnight in SC-Ura-His medium containing 2% (w/v) galactose at 30°C. Cultures were then diluted to OD_600_ of 0.4. After 12 h, cultures were serial diluted as previously described above, and 10 μl of each dilution was plated onto a SC-His plate with 2% (w/v) glucose and 1% (w/v) 5-FOA. In addition, as a control, 10 μl of each dilution was plated onto a SC-Ura-His plate with 2% (w/v) galactose. The plates were incubated at 30°C for 72 h.

### Yeast Lipid Analysis

*Saccharomyces cerevisiae* mutant strain ALE1 was transformed with the pYES2 constructs containing the sunflower *LPCAT* genes, and also co-transformed with p416GPD:*Cs*FAD2, p423GPD:*Ha*LPCATs and the appropriated empty plasmids (Mumberg et al., 1995). The cultures were grown for 24 h in SC-Ura-His medium supplemented with 2% (w/v) glucose and then diluted to an OD_600_ of 0.4. The yeasts were incubated for 48 h and then diluted to an OD_600_ of 4 in 200 ml of medium. Cells were collected by centrifugation and washed twice with distilled water. The total lipids were extracted (Bligh and Dyer, 1959), evaporated under nitrogen, and dissolved in 1 ml chloroform.

Glycerolipid species were determined by high performance liquid chromatography analysis as described by Salas et al. (2006).

To determine the fatty acid composition of yeast lipid species, the different lipid species were separated by thin layer chromatography and their fatty acid composition determined according to Álvarez-Ortega et al. (1997).

### Acyl-CoA Pool Analysis

For acyl-CoA determination, the optical density yeast cultures were adjusted with fresh medium and 50 ml of culture was centrifuged and washed with distilled water; 1 nmol of heptadecanoyl-CoA was added as an internal standard and acyl-CoAs were extracted from transformed yeast and derivatized to their acyl-etheno derivatives, as indicated by Larson and Graham (2001). Briefly, the optical density of the cultures was adjusted with fresh medium and 50 ml of culture was centrifuged and washed with distilled water. Cells were then pelleted by centrifugation at 10,000 × *g* for 15 min and 1 nmol of heptadecanoyl-CoA was added an internal standard. The cell pellets were lysed by vortexing with 250 μl of freshly prepared extraction buffer: 2 ml 2-propanol, 2 ml KH_2_PO_4_ 25 mM pH 7.2, 50 μl glacial acetic acid, and 80 μl BSA (50 mg/ml) in the presence of 1 ml of glass beads (710–1180 μm; Sigma–Aldrich, United States). The lipids were then extracted and the protein was precipitated by adding 600 μl methanol/chloroform 2:1 v/v. The tubes were incubated for 20 min, and centrifuged at 13,000 × *g* for 2 min. The aqueous supernatants were dried under nitrogen and dissolved in 300 μl chloroacetaldehyde reagent. The derivatization reaction was carried out at 85°C for 20 min in dark. Acyl-CoA etheno derivatives were analyzed in a Waters 2695 separation module endowed with a XBridge C18 column (250 × 0.5 mm, 5 μm; Waters) and a Multi λ Fluorescence Detector 2475 (Waters) using a modified version of the quaternary gradient system described by Larson and Graham (2001). The solvents consisted of A: 1% acetic acid; B: 90% acetonitrile, 1% acetic acid; C: 0.25%, trimethylamine, 0.1% tetrahydrofurane; and D: 90% acetonitrile. The flow was 0.75 ml/min, and the analysis temperature 40°C. The gradient elution profile was comprised the following steps: 0–7.0 min, A–B 90:10 to A–B 20:80; 7.0–7.1 min, A–B 20:80 B to A–C 20:80; 7.1–9.0 min, A–C 20:80 to C–D 90:10; 9.0–34.0 min, C–D 90:10 to C– D 25:75; 34.0–35.0 min, C–D 25:75 to D 100; 35.0–39.0 min, D 100; 39.0–40.0 min, D 100 to A–B 90:10; 40.0–45.0 min, A–B 90:10. The detector was set with an excitation wavelength of 230 nm and a detection one of 420 nm. Peaks were quantified attending to the internal standard signal. Data were processed using the Empower Login software.

### Microsomal Membrane Preparation From Yeast

*slc1*Δ yeast cultures transformed with different pYES2 constructs were grown in SC-Ura medium supplemented with 2% (w/v) raffinose for 3 days. The cultures were then diluted to an OD_600_ of 0.4 in SC–Ura medium supplemented with 2% (w/v) galactose and 1% (w/v) raffinose. After 24 h of induction, stationary cultures were diluted to an OD_600_ of 4 in 200 ml of medium. Then, cells were pelleted by centrifugation and washed twice with distilled water for the preparation of microsomes according to the method described in Dahlqvist et al. (2000). Protein in microsomal fraction was measured using the BCA method, using the kit from Thermo Scientific.

### LPCAT Assay

Radiolabeled *sn-*1–18:1-*sn-*2-[1-^14^C]-acyl-PC and [1-^14^C]-18:1-LPC were prepared according to the method described by Kanda and Wells (1981) from commercial radioactive fatty acids. The forward and reverse reactions catalyzed by LPCAT were assayed according to Lager et al. (2013). The activity was measured by monitoring the formation of radiolabeled PC from [1-14C]-18:1-LPC (1 μCi/μmole) and different non-labeled commercial acyl-CoAs.

For the reverse reaction, three different species of radiolabeled PC were used; all of them were prepared from *sn*-1-18:1-LPC, which was acylated with [1-^14^C]-18:1, [1-^14^C]-18:2, and [1-^14^C]-18:3. The activity was determined by measuring the radiolabeled acyl-CoA released in the presence of free CoA (Lager et al., 2013). In both cases, control reactions were run at time zero. The counting obtained in control reactions was subtracted to calculate enzyme activity.

### Plant Transformation and Molecular Analysis of Transgenic Events

Sequences of open reading frames (ORFs) of three sunflower *LPCATs* (*HaLPCAT1*, *2*, and *3*) were cloned in the pBIN19:35S binary plasmid (Sayanova et al., 1997). These constructs were transferred to *Agrobacterium tumefaciens* strain GV3101 and kanamycin-resistant colonies were selected in all cases. Arabidopsis double mutant, Col-0, or null segregants were transformed with these constructs by the floral dipping method (Bechtold et al., 1993; Clough and Bent, 1998). Transgenic plants were confirmed by amplification of genomic DNA extracted according to Kasajima et al. (2004) method. And finally, the gene expression in transgenic plants was confirmed by PCR analysis of RNA from old leaves. In both cases, specific primers were designed based on construct sequence (Supplementary Table S1).

Oil content and fatty acid composition of Arabidopsis seed was determined by transmethylating lipids and analyzing fatty acid methyl esters of 50 seeds after the addition of 60 μg of 17:0 internal standard as described by Garcés and Mancha (1993).

### Statistics Analysis

To stablish significant differences between determinations, one-way ANOVA was used combined with Tukey *post hoc* analysis with a significance level of 0.05%. The calculations were performed using program Sigma Plot 14.0 (Systat Software Inc.).

## Results and Discussion

### Cloning of Three Putative Sunflower LPCATs and Sequence Analysis

Plant lipid metabolism is a highly dynamic process, often involving acyl exchange between the different species of glycerolipids and other intermediates (Chapman and Ohlrogge, 2012). Within this metabolic context, LPCATs play an important role in catalyzing acyl exchange between PC and acyl-CoA pools. They have been demonstrated to be involved in the incorporation of *de novo* synthesized acyl moieties into glycerolipids through the acyl editing pathway and the redistribution of PUFAs from PC to other glycerolipids, including reserve TAG (Bates et al., 2012). In the present work, three genes coding for sunflower LPCAT were cloned from cDNA obtained from developing sunflower seed mRNA by PCR using specific primers (Supplementary Table S1). Two complete EST, corresponding to sunflower LPCATs, were found in the NCBI database using TBLASTN program and the Arabidopsis LPCAT protein sequence. These sequences were then used to search in the sunflower genome platform and a third gene was identified and cloned using the same procedure. The three genes were named *HaLPCAT1*, *HaLPCAT2*, and *HaLPCAT3*, which were deposited in the Genbank as JN112899, JN112900, and KY235263, respectively. These sequences correspond to references HanXRQChr13g0416721 (*HaLPCAT1*), HanXRQChr02g0048801 (*HaLPCAT2*), and HanXRQChr17g0533761 (*HaLPCAT3*) within sunflower genome^5^. They coded for putative proteins of 462, 459, and 463 amino acid residues, with corresponding molecular masses of 51.88, 51.56, and 52.37 kDa, respectively. The proteins were markedly alkaline, with pI values of 9.36, 9.40, and 9.51 for the three isoforms, respectively. The three peptides were similar to each other, with degrees of identities ranging from 73 to 83% in their amino acid sequences. These sequences were aligned with those from other species (Supplementary Figure S1) and displayed high levels of identity and homology with other forms previously described in monocots, dicots, and moss (Supplementary Figure S1). They displayed a high level of identity with very few gaps, even though a large taxonomic range of species was included (Supplementary Figure S1). This pointed to a high degree of conservation among these enzymes along the period of evolution of the species investigated. When these proteins were characterized using the CDD (Marchler-Bauer et al., 2017), they were classified within the membrane-bound O-acyltransferase (MBOAT) family, a group of membrane-bound enzymes that catalyzes the transfer of acyl-moieties to various acyl acceptors. In all cases, the two highly conserved polar amino acid residues necessary for catalysis in these enzymes were located as His356 and Asn318 (Supplementary Figure S1). In this regard, Zhang et al. (2015) identified two other conserved amino acid residues in plants, Ser130 and Leu325, participating in catalysis in the *Ha*LPCATs. Furthermore, the four motifs: A (WD), B (WHGxxxGYxxxF), C (YxxxxF), and D (YxxxYFxxH), described for plant lysophospholipid acyltransferase enzymes in the MBOAT family were also localized in the three isoforms isolated from sunflower. Motif B is highly conserved in LPCATs, while motifs A, C, and D are also present in other acyltransferases related to fatty acid metabolism, which could be involved in binding to the lysophospholipid substrate. Therefore, LPCATs belong to a large and diverse superfamily of proteins known as the lysophospholipid acyltransferases (LPLATs) (Shindou et al., 2009).

Sunflower LPCATs, like the ones from other plant species, are enzymes very strongly associated to membranes. The program Top Pred II (Claros and Heijne, 1994) predicted the presence of nine transmembrane domains in these proteins (Supplementary Figure S2) using the hydrophobicity scale KD (Kyte and Doolittle, 1982), indicating they are integral membrane proteins. The C-terminal domains of the *Ha*LPCAT proteins display a di-lysine motif corresponding to a signal of retention in the ER. This location was according with the subcellular location prediction program DeepLoc (Almagro Armenteros et al., 2017; Supplementary Figure S3), although experimental evidence is still pending to confirm the exact location of these enzymes.

### Phylogenetic Analysis of Sunflower LPCAT Enzymes

**FIGURE 1:**
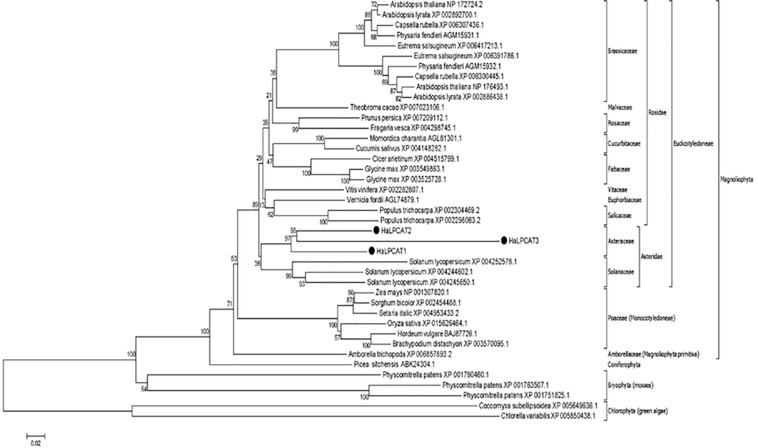
Phylogenetic tree of different plant LPCAT proteins, including sunflower HaLPCAT1, 2, and 3. The tree was rooted with proteins from green algae (Chlorophyte). The groups and species included dicots, monocots, and bryophytes. The black dots indicate LPCATs from sunflower.

Amino acid sequences of the *Ha*LPCATs were used to study their phylogenetic relationship with other homologs described in plant species. The neighbor-joining method from MEGA 6 (Tamura et al., 2013) was used to construct a phylogenetic tree using green algae (*Chlorophyte*) as an external group for tree rooting (Figure 1). The LPCAT enzymes are broadly distributed within the plant kingdom, although the sequences of LPCATs from the species studied appeared highly conserved during the evolutionary period analyzed, displaying low functional diversification, which pointed again to an important role in evolutionary adaptation. The phylogenetic tree shows that LPCATs from dicotyledonous plants form a large clade, suggesting a common origin. The tree is compatible with an early differentiation of Chlorophyta and Bryophyta from monocotyledonous and dicotyledonous plants. The *Ha*LPCATs proteins displayed a close relationship with those from tomato (*Solanum lycopersicum*). This was not unexpected since tomato shares a subclass with sunflower (*Asteridae*).

### Analysis of Gene Expression of HaLPCATs

**FIGURE 2:**
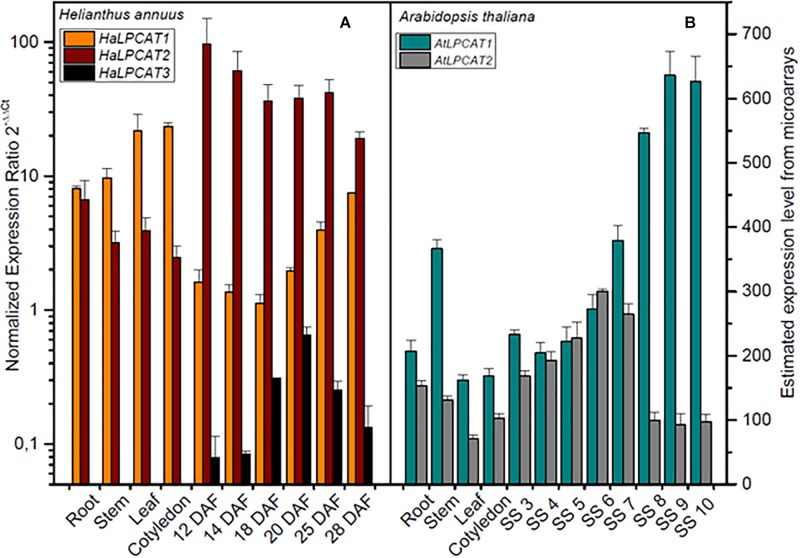
Expression levels of *LPCATs* sunflower **(A)** and Arabidopsis **(B)** in developing seeds and vegetative tissues. Sunflower genes expression was determined by qRT-PCR using the actin gene from *H. annuus* (GenBank FJ487620) as a reference gene. The data correspond to the mean±SD from three independent measurements. *Arabidopsis thaliana LPCAT* gene expression was taken from microarray data (Schmid et al., 2005): DAF, days after flowering; SS 3, mid-globular to early heart embryos; SS 4, early to late heart embryos; SS 5, late heart to mid-torpedo embryos; SS 6, mid to late torpedo embryos; SS 7, late torpedo to early walking-stick embryos; SS 8, walking-stick to early curled cotyledons embryos; SS 9, curled cotyledons to early green cotyledon embryos; SS 10, dry seeds.

The expression patterns of the sunflower *LPCAT* genes were examined by qRT-PCR on cDNA extracted from different plant tissues, involving, roots, stem, leaf, cotyledons, and developing seeds from 12 to 28 DAF. In all cases, the amplification efficiency was between 92 and 110%, being the regression coefficients > 0.985 (Supplementary Table S2). The profiles of expression of the three genes are shown in Figure 2A. *HaLPCAT1* and *HaLPCAT2* were ubiquitously expressed in all tissues; the *HaLPCAT2* gene displayed the highest expression level in developing seeds during the period of oil accumulation, which occurs from 18 to 30 DAF (Aznar-Moreno et al., 2013). The *HaLPCAT1* gene, however, was expressed at higher levels in vegetative tissues (root, leaf, and stems) and germinating cotyledons, although it also displayed a substantial level of expression in developing seeds. The expression profiles of these two forms were quite similar to that displayed by the *AtLPCAT1* and *AtLPCAT2* in Arabidopsis (Figure 2B). The ubiquitous expression of *LPCATs* have also been observed in other plant species including *Nicotiana benthamiana*, *B. napus*, and *Ricinus communis* (Schmid et al., 2005; Wang et al., 2012; Zheng et al., 2012; Arroyo-Caro et al., 2013; Zhang et al., 2015). In the case of sunflower, a third form of *LPCAT* was also present. The *HaLPCAT3* gene was specific to developing seeds, with no detectable transcription occurring in cotyledons or vegetative tissues. The level of expression of this gene, however, was by far the lowest of the three genes in those tissues. It is remarkable that no seed specific LPCAT forms have been previously reported in other plants. Nevertheless, the relatively high expression of *HaLPCATs* in developing seeds is consistent with the fact that high LPCAT activity is necessary to support the acyl flow requirements associated with accumulation of seed TAGs containing PUFAs.

### Assays of *in vivo* Complementation of Yeast Mutant

**FIGURE 3:**
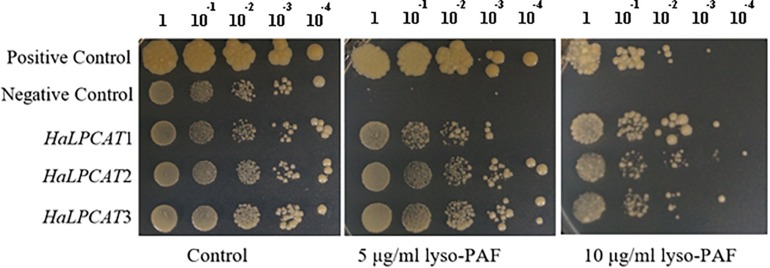
Lyso-platelet activating factor (PAF) sensitivity test for *Saccharomyces cerevisiae* mutant Y02431 (ALE1 or *slc4*△) expressing sunflower *LPCATs* or harboring the pYES2 empty vector (negative control) and S288C yeast strain (positive control). Yeast cells were grown overnight and induced for protein expression for 24 h were diluted 1:10 from DO_600_ = 2.0 to DO_600_ = 2 × 10^−4^. The resulting 10 μl yeast suspension was spotted on a SC-Ura agar plate containing 5 and 10 μg/ml lysoPAF. The growth of yeast cells was evaluated after 72 h at 30°C.

The heterologous expression of *HaLPCATs* was carried out in yeast, a host that has been demonstrated to be appropriate for the characterization of other ER-associated enzymes involved in lipid metabolism (Zheng et al., 2012; Rodríguez-Rodríguez et al., 2016; González-Mellado et al., 2019). The first step in characterizing the recombinant sunflower LPCATs, produced in yeast, was to assess their functionality *in vivo*. Sequence analysis of the clones indicated that they corresponded to complete ORFs and should be active enzymes. This point was demonstrated through assays of complementation of the *slc4* yeast mutant (Y02431), defective in lysophospholipid acyltransferase activity. Although the *slc4* mutant was shown to be viable, it has also been reported to be hypersensitive to choline-containing lysolipids and lyso-PAF (Zaremberg and McMaster, 2002). The production of an active LPCAT makes it possible to acylate lyso-PAF, which is then incorporated into membranes in a non-toxic manner. This assay has been used previously to assess functionality of LPCATs from other sources including *B. napus* and *Nicotiana benthamiana* (Chen et al., 2007; Zheng et al., 2012; Zhang et al., 2015). Lyso-PAF sensitivity tests for the yeast mutant Y02431, expressing sunflower *LPCAT* cDNAs were run to assess if they encoded functional enzymes. The assay consisted of growing a control strain (S288C), the mutant Y02431 expressing the different sunflower *LPCAT* cDNAs and harboring the pYES2-Ura empty vector at increasing concentrations of lyso-PAF ether analogous to LPC. Control yeast lines were able to grow in the presence of lyso-PAF, whereas the mutant Y02431, which is unable to acylate LPC due to a mutation in the gene *SLC4*, was very sensitive to the presence of that compound in the medium (Figure 3). The expression of each sunflower *LPCAT* cDNA in that mutant was able to complement the mutation, making the yeast strain able to grow in the presence of lyso-PAF. This result demonstrated that all these genes encode functional LPCATs that can be recombinantly produced in yeast and take part in the lipid metabolism of the host cells.

**FIGURE 4:**
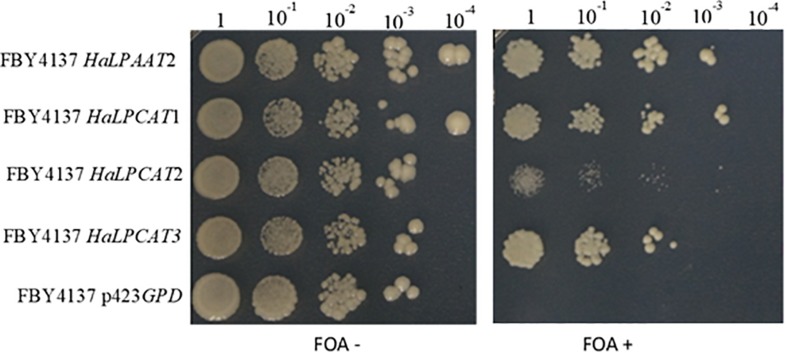
Complementation of the slc1Δslc4(Δ Saccharomyces cerevisiae mutant FBY4137 harbouring pGREG546:*Sc*SLC1) with the three HaLPCAT isoforms, using 5-fluoroorotic acid (FOA) as a selective agent. The strains are displayed in rows and the dilutions in the columns. The LPAAT2 gene from sunflower was used as a positive control.

Another aspect of LPCATs that can be investigated by complementation assays is the capacity of the enzymes to acylate substrates different from PC. In this regard, previous studies pointed to the capacity of these enzymes to acylate LPA and thus also contribute to *de novo* glycerolipid synthesis. The strategy to probe this aspect involved a counter selection trial using the *S. cerevisiae* strain FBY4137. This line contains the double mutation *slc1*Δ/*slc4*Δ and is unable to acylate LPA (Benghezal et al., 2007). This double mutation is lethal, but the mutant can grow when harboring the pGREG546:*Sc*SLC1 plasmid, which also expressed the URA3 protein. When the medium is supplemented with 5-FOA, the enzyme URA3 converts that compound in toxic 5-fluorouracil. So, the only means of the yeast to grow on 5-FOA is to lose the pGREG546:*Sc*SLC1 plasmid. If another enzyme which is able to acylate LPA is present, the yeast would grow in that medium. An assay of counter-selection in the presence of 5-FOA was run to investigate if the HaLPCATs also displayed LPAAT activity. In these trials, the lethal double mutant strain FBY4137 (*slc1*Δ/*slc4*Δ) harboring the pGREG546:*Sc*SLC1 plasmid was transformed with constructs expressing *HaLPCAT* genes in the p423GPD plasmid. In a medium containing 5-FOA, yeast have to lose the pGREG546:*Sc*SLC1 plasmid expressing orotidine 5′-phosphate decarboxylase gene (URA3) to survive. Thus, yeast will only be viable if the other plasmid encodes an active LPAAT. The results of these assays are shown in Figure 4. In the absence of 5-FOA, all yeast lines were viable. When the reactant was added to the medium, the FBY4137 cells carrying the empty p423*GPD* plasmid were not viable, whereas the mutants producing a functional LPAAT from sunflower were able to grow. The expression of all the *HaLPCAT* cDNAs studied in this work was able to complement the FBY4137 mutant, so the three yeast lines growing under the conditions were viable (Figure 4). This demonstrated that these enzymes were able to acylate LPA and participate in the *de novo* glycerolipid synthesis. This was also observed for LPCAT isoforms from Arabidopsis, *B. napus*, *N. benthamiana*, and *R. communis* (Ståhl et al., 2008; Zheng et al., 2012; Arroyo-Caro et al., 2013; Zhang et al., 2015). It was also remarkable that the yeast expressing *HaLPCAT2* grew slower than those harboring *HaLPCAT1* or *HaLPCAT3*, which suggests a distinct functionality for *Ha*LPCAT2.

### Impact of Recombinant HaLPCAT Production on Yeast Lipid Composition

Yeast transformed with *HaLPCAT*s were induced and grown to the stationary phase. Then, lipids were extracted and analyzed by high performance liquid chromatography and gas liquid chromatography. To examine the effect of LPCATs on the metabolism of PUFAs, two series of experiments were run. In the first series, in mutant Y02431, only *HaLPCATs* were expressed. In the second series, a microsomal *FAD2* from *C. sativa* (Rodríguez-Rodríguez et al., 2016) was co-expressed together with each of the *HaLPCATs*. *HaLPCATs* were expressed using the p423GPD vector, whereas *CsFAD2* was carried in the p416GPD vector. Control cells were transformed with the corresponding empty plasmids. The method used allowed the quantification of the majority of neutral and polar glycerolipid species present in yeast, involving TAG, CL, PE, PC, PS, and PI. The main focus was on PE and PC since these were the species most directly involved in the LPCAT reaction. The total amount of polar glycerolipids did not significantly change in the yeast lines investigated, remaining in the range from 23.3 to 39.0 μg/ml culture (Table 1). The relative composition of the different glycerolipids remaining was less variable. In the first set of samples (with no *CsFAD2* expression), only small changes were observed. The only significant differences were observed in the contents of PC and PI when *HaLPCAT2* was expressed, involving a decrease of the former species at expense of an increase of the later one (Table 1). This change was difficult to interpret since PI and PC synthesis are not directly interconnected. PI is produced from DAG-CDP, which has PA as its precursor whereas the main precursor of PC is DAG (Henry et al., 2012). The increase in PI content might be a stress response to the expression of the enzyme, which could in some manner alter yeast membrane properties. The involvement of PI and PI-related metabolism on yeast stress responses has been previously reported for ethanol resistance and osmotic stress (Chi et al., 1999; Bonangelino et al., 2002). Another explanation for this could be that the LPCATs investigated displayed activity with

**TABLE 1:** Polar lipid content and composition from *Saccharomyces cerevisiae* strain Y02431 (*slc4*Δ) expressing *LPCATs* from sunflower in the absence or presence of expression of a *FATTY ACID DESATURASE* (*FAD2*) from *Camelina sativa*.

		Total content			Mole %		
		(μ g/ml culture)	CL	PE	PC	PS	PI
*CsFAD2*−	p423GPD p416GPD	23.3 ± 7.2	1.3 ± 0.6	14.1 ± 3.0	54.4 ± 2.5	5.8 ± 0.9	24.4 ± 5.7
	p423GPD:*HaLPCAT1* p416GPD	33.1 ± 4.5	2.1 ± 1.2	15.0 ± 1.9	53.7 ± 2.1	6.1 ± 1.8	23.2 ± 3.4
	p423GPD:*HaLPCAT2* p416GPD	32.2 ± 5.9	1.0 ± 0.2	11.5 ± 1.4	47.1 ± 1.7*	7.9 ± 2.6	32.5 ± 1.9*
	p423GPD:*HaLPCAT3* p416GPD	24.4 ± 5.1	1.4 ± 0.4	20.5 ± 1.1*	52.9 ± 1.3	4.3 ± 0.4	20.9 ± 2.1
*CsFAD2*+	p423GPD p416GPD:CsFAD2	29.3 ± 8.5	1.0 ± 0.4	15.0 ± 4.1	48.4 ± 5.7	5.1 ± 1.2	30.5 ± 9.3
	p423GPD:*HaLPCAT1* p416GPD:CsFAD2	39.0 ± 13.7	1.1 ± 0.4	12.3 ± 2.7	48.2 ± 3.5	4.9 ± 0.7	33.6 ± 3.0
	p423GPD:*HaLPCAT2* p416GPD:CsFAD2	29.9 ± 13.1	1.2 ± 0.6	15.7 ± 2.9	60.9 ± 4.9*	5.1 ± 0.6	17.1 ± 2.9*
	p423GPD:*HaLPCAT3* p416GPD:CsFAD2	27.9 ± 3.3	1.9 ± 0.8	19.5 ± 0.7	53.9 ± 2.4	4.3 ± 0.3	20.3 ± 1.3

*HaLPCATs were expressed in the vector p423GPD, whereas *CsFAD2* was expressed in p416GPD. Control lines correspond to empty plasmids. Data are the average of four independent determinations ± standard deviation. Asterisks indicate significant differences with control cells according to one-way ANOVA with Tukey *post hoc* analysis with a significance level of 0.05%. CL: cardiolipin; PE: phosphatidylethanolamine; PC: phosphatidylcholine; PS: phosphatidylserine; PI: phosphatidylinositol.*

other species of phospholipids. This was supported by results in Figure 4, which demonstrated certain activity toward LPA. Furthermore, a significant slight increase of PE was also observed when *HaLPCAT3* was expressed (Table 1), which pointed that this form could have lysophosphatidyl ethanolamine:acyl-CoA acyltransferase (LPEAT) activity. Previous works on *A*tLPCATs showed they displayed activity with other phospholipid species. Thus, Ståhl et al. (2008) assayed *S. cerevisiae* LPAT, *At*LPCAT1 and *At*LPCAT2 for acylation of different lysophospholipids including LPC, LPA, lysophosphatidyl ethanolamine (LPE), lysophosphatidyl glycerol (LPG), lysophosphatidyl serine (LPS), and lysophosphatidyl inositol (LPI). *S. cerevisiae* LPAT displayed broad substrate specificity, being especially active toward LPC and LPS. Arabidopsis LPCATs were on the contrary more specific for LPC, although they were clearly able to acylate LPE, LPG, and LPS at a rate around fourfold lower than LPC. Only traces of activity were found for LPI. A later study reproduced those results (Jasieniecka-Gazarkiewicz et al., 2016) and made comparison of profiles with an *At*LPEAT, which clearly displayed higher activities at acylating LPE. In the case of sunflower LPCATs, the activity toward LPA was demonstrated in the complementation assays. Results in Table 1, in which yeast phospholipids were differently affected by the expression of the different *Ha*LPCAT forms, pointed those enzymes were also able to acylate other lysophospholipid species like LPE or even LPI. *In vitro* studies in this regard could be an interesting field for future research.

Thus, although *Ha*LPCATs displayed large degree of identity, they seemed to act differently when produced in the yeast host. In the second series of samples (expressing *CsFAD2*), the only significant differences were also found in yeasts transformed with *HaLPCAT2* and in the same polar lipid species, PC and PI, but in this case, the variation was the opposite, and an increase of PC was observed accompanied by a decrease of PI (Table 1). In this case, the increase in ER membrane fluidity caused by the presence of linoleate might have been a contributing factor.

**FIGURE 5:**
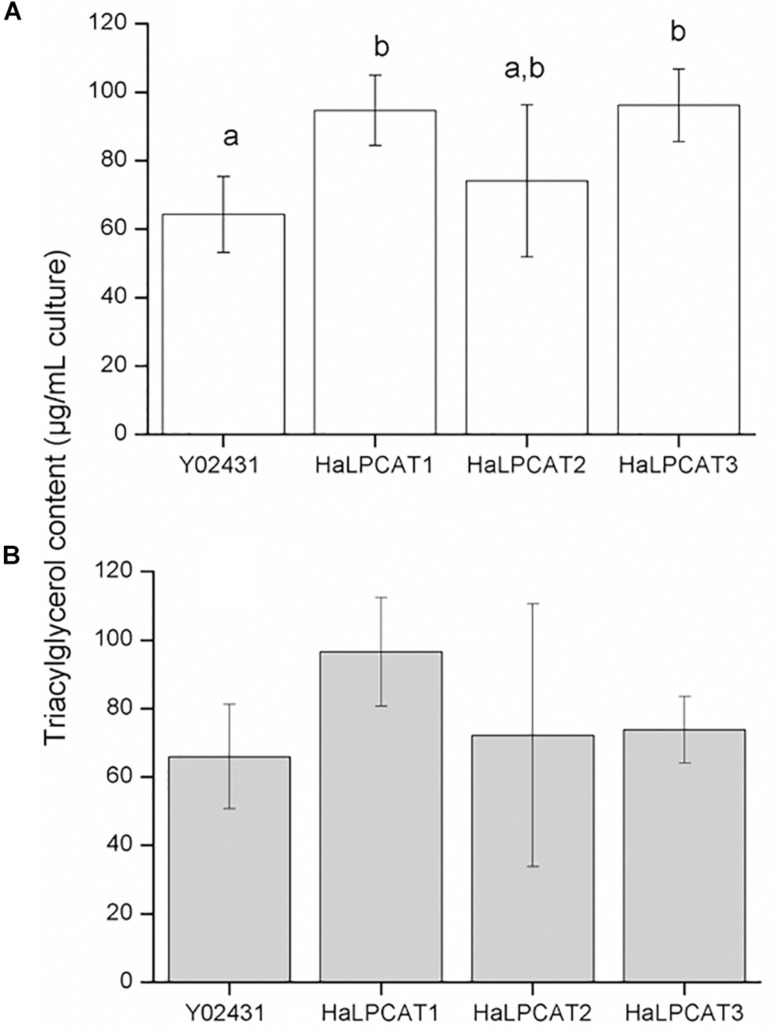
Triacylglycerol content of *Saccharomyces cerevisiae* strain Y02431 (ALE1) expressing sunflower *LPCATs* in the absence **(A)** or presence of *Camelina sativa* fatty acid desaturase 2 expression **(B)**. Data represent the average of four independent preparations,±standard deviation. The letters indicated the groups of statistical significance according to a one-way ANOVA with Tukey *post hoc* analysis at a significance level of 0.05%.

The effect of recombinant *Ha*LPCAT production on yeast TAG fraction was also investigated. When the experiment was run in the absence of expression of *CsFAD2*, a significant increase in TAG content was observed in the cultures expressing *HaLPCAT1* or *HaLPCAT3*, which rose from 60 to 90 μg TAG/mL culture, a 50% increase (Figure 5A). This increase was not observed with *HaLPCAT2* expression. When *CsFAD2* was co-expressed with *HaLPCATs*, the effect of LPCAT activity on the TAG content was not so obvious (Figure 5B). In this regard, a slight but insignificant increase in TAG content was observed when *HaLPCAT1* was expressed. The increase in TAG content caused by the production of *Ha*LPCAT1 or *Ha*LPCAT3, in the absence of *CsFAD*2 expression, might be associated with increased acylation of LPA due to the LPAAT activity of these *Ha*LPCATs. These results support the hypothesis that increasing the production of acyltransferases in yeast leads to a greater flux of intermediates and results in enhanced TAG accumulation, as described in plants (Zou et al., 1997; Woodfield et al., 2019). Indeed, yeast expressing *HaLPCAT1* or *HaLPCAT3* grew faster than yeast expressing *HaLPCAT2* in the LPAAT activity assessment assays (Figure 4). The phosphatidate produced by LPAAT action leads to DAG that can be used for production of both nitrogenous glycerolipids and TAG (Kennedy, 1961). The ability of *Ha*LPCAT1 or *Ha*LPCAT3 to catalyze the acylation of LPA may have been diminished in yeast co-expressing *CsFAD2* due to alterations in membrane fluidity associated with increase linoleate content of the ER.

### Changes Induced by Recombinantly Produced HaLPCAT in Yeast Glycerolipids Fatty Acid Composition

The fatty acid composition of the of PC, PE, and TAG was investigated in yeast producing recombinant *HaLPCATs* and *CsFAD2* in experiments performed in a similar way to the previous study. The changes induced by the production of *Ha*LPCATs on the fatty acid composition of the mentioned lipid species from the Y02431 yeast strain are shown in Table 2. In the absence of *C*s*FAD2* expression, the fatty acids identified were those typically found in *S. cerevisiae*, with Δ9 and Δ11 fatty acids being the only unsaturated forms. The expression of *HaLPCAT*s impacted mainly in the content of *n-7* fatty acids (16:1^Δ^^9^ and 18:1^Δ^^11^), resulting in a decrease in the proportion of 18:1^Δ^^11^ in all lipid species. This fatty acid is produced by elongation of 16:1^Δ^^9^-CoA in an Elo1p-dependent manner (Schneiter et al., 2000). *Ha*LPCAT action may have influenced the turnover of 16:1 such that the availability of that substrate was reduced. Furthermore, *Ha*LPCAT1 production induced significant increases of 16:1 in PC and TAG at expense of 16:0. This form also increased the content of 18:1^Δ^^9^ in PC, suggesting that this enzyme isoform contributed to the exchange of monounsaturated fatty acids between PC and the acyl-CoA pool (Table 2). In contrast, in the case of *Ha*LPCAT2, its effect seemed to be the opposite to *Ha*LPCAT1, causing increases in the content of 16:0 and decreases in 16:1 in PC and PE, but not affecting TAGs.

**TABLE 2:** Fatty acid composition of triacylglycerols (TAG), phosphatidylethanolamine (PE) and phosphatidylcholine (PC) from *Saccharomyces cerevisiae* strain Y02431 (*slc4*Δ) expressing *LPCATs* from sunflower in the presence or absence of expression of *Camelina sativa FATTY ACID DESATURASE 2* (*CsFAD2*).

Composition (mole%)										
*CsFAD2*−		16:0	16:1^Δ9^	16:2^Δ9,12^	18:0	18:1^Δ9^	18:1^Δ11^	18:2^Δ9,12^	18:2^Δ11,14^	
PC	Control	38.6 ± 5.3	21.2 ± 2.8	*nd*	13.3 ± 2.0	19.7 ± 3.5	7.2 ± 2.3	nd	nd	
	*HaLPCAT1*	24.9 ± 1.4*	31.6 ± 1.8*	nd	11.3 ± 1.4	27.6 ± 1.4*	4.6 ± 1.1*	nd	nd	
	*HaLPCAT2*	61.1 ± 1.3*	8.1 ± 1.4*	nd	13.3 ± 2.0	12.9 ± 2.0	4.7 ± 4.3*	nd	nd	
	*HaLPCAT3*	28.4 ± 5.7	26.9 ± 6.1	nd	15.9 ± 3.7	24.2 ± 3.8	4.7 ± 1.2*	nd	nd	
PE	Control	45.5 ± 0.4	18.4 ± 4.8	nd	12.8 ± 8.5	18.3 ± 1.8	4.9 ± 1.5	nd	nd	
	*HaLPCAT1*	47.9 ± 4.1	21.0 ± 1.7	nd	13.3 ± 4.7	15.2 ± 2.9	2.7 ± 0.3*	nd	nd	
	*HaLPCAT2*	61.9 ± 1.3*	8.1 ± 1.4*	nd	13.5 ± 2.0	14.4 ± 2.0	2.3 ± 1.1*	nd	nd	
	*HaLPCAT3*	42.6 ± 3.9	17.6 ± 4.4	nd	22.1 ± 9.4	15.0 ± 1.8	2.8 ± 0.4*	nd	nd	
TAG	Control	20.9 ± 1.0	42.8 ± 1.4	nd	9.2 ± 0.4	19.1 ± 1.5	8.0 ± 1.3	nd	nd	
	*HaLPCAT1*	17.1 ± 3.4	52.0 ± 5.3*	nd	6.3 ± 1.5	20.3 ± 2.2	4.4 ± 0.9*	nd	nd	
	*HaLPCAT2*	23.1 ± 7.4	41.1 ± 4.7	nd	9.5 ± 2.1	22.0 ± 4.5	4.3 ± 1.1*	nd	nd	
	*HaLPCAT3*	19.8 ± 2.0	42.6 ± 0.7	nd	10.9 ± 0.8	22.0 ± 2.1	4.6 ± 1.0*	nd	nd	

***CsFAD2*+**		**16:0**	**16:1^Δ9^**	**16:2^Δ9,12^**	**18:0**	**18:1^Δ9^**	**18:1^Δ11^**	**18:2^Δ9,12^**	**18:2^Δ11,14^**	**Total PUFA**

PC	Control	45.1 ± 8.1	15.7 ± 4.9	2.2 ± 0.9	20.2 ± 2.4	6.9 ± 1.7	1.8 ± 0.5	7.8 ± 3.5	0.3 ± 0.1	10.4 ± 3.6
	*HaLPCAT1*	33.8 ± 3.8*	21.9 ± 6.6	3.5 ± 1.4	19.5 ± 5.3	7.1 ± 2.0	1.5 ± 0.5	12.1 ± 3.2	0.5 ± 0.2	16.1 ± 3.5
	*HaLPCAT2*	51.6 ± 4.9	8.7 ± 4.0	0.8 ± 0.2	28.4 ± 2.5	5.7 ± 2.8	1.3 ± 0.6	3.3 ± 0.2	0.2 ± 0.0	4.2 ± 0.3
	*HaLPCAT3*	31.6 ± 1.3*	25.4 ± 1.5*	2.5 ± 1.0	16.5 ± 0.8	9.0 ± 1.9	4.6 ± 0.4*	10.0 ± 0.6	0.5 ± 0.1	13.0 ± 1.2
PE	Control	76.3 ± 4.2	5.5 ± 2.3	0.0 ± 0.0	14.5 ± 2.7	3.3 ± 0.3	0.0 ± 0.0	0.4 ± 0.1	0.0 ± 0.0	0.4 ± 0.1
	*HaLPCAT1*	50.8 ± 6.1*	16.5 ± 2.4*	1.5 ± 0.4*	14.3 ± 2.2	6.4 ± 2.4	0.5 ± 0.7	10.0 ± 1.1*	0.0 ± 0.0	11.5 ± 1.2*
	*HaLPCAT2*	72.5 ± 4.4	6.2 ± 1.8	0.0 ± 0.0	16.3 ± 2.5	2.7 ± 3.4	0.5 ± 0.7	1.8 ± 1.2	0.0 ± 0.0	1.8 ± 1.2
	*HaLPCAT3*	53.1 ± 4.0*	21.2 ± 2.1*	0.4 ± 0.1*	7.9 ± 2.7*	11.7 ± 3.6*	1.9 ± 0.7*	3.8 ± 0.6*	0.0 ± 0.0	4.2 ± 0.6*
TAG	Control	20.8 ± 3.8	37.5 ± 4.5	5.5 ± 1.4	9.2 ± 1.5	11.5 ± 1.8	3.2 ± 1.0	11.6 ± 2.4	0.6 ± 0.3	17.8 ± 2.8
	*HaLPCAT1*	18.5 ± 3.2	32.6 ± 5.3	8.0 ± 2.3	10.3 ± 1.1	9.5 ± 1.8	1.9 ± 0.5*	18.2 ± 3.8*	1.0 ± 0.4	27.2 ± 4.4*
	*HaLPCAT2*	22.7 ± 3.7	35.0 ± 7.2	4.6 ± 2.0	12.1 ± 3.8	11.0 ± 2.8	3.1 ± 0.8	10.9 ± 1.1	0.5 ± 0.2	16.1 ± 2.3
	*HaLPCAT3*	19.0 ± 2.2	36.9 ± 4.2	6.2 ± 1.5	9.9 ± 1.5	9.2 ± 0.9	4.8 ± 1.3	13.1 ± 1.4	0.9 ± 0.2	20.2 ± 2.1

*Data are the average of four independent determinations ± standard deviation. Asterisks indicate significant differences with control cells according to one-way ANOVA with Tukey post hoc analysis with a significance level of 0.05%. nd: not detected.*

The expression of *CsFAD2* increased the complexity of yeast fatty acid composition (Table 2). PUFA species appeared as consequence of *Cs*FAD2 activity (Rodríguez-Rodríguez et al., 2016), resulting in 18:2^Δ9,12^ and 16:2^Δ9,12^, and small amounts of 18:2^Δ11,14^, presumably produced by elongation of 16:2^Δ9,12^ (Table 2). In control yeast, the PUFAS produced were mainly present in PC and TAG, with very little incorporation into PE molecules. Expression of *CsFAD2* induced a considerable increase in the proportion of saturated fatty acids in PE species. This may have been related physiological regulation of the fluidity of the ER in yeast. This effect on yeast lipids was previously reported by Rodríguez-Rodríguez et al. (2016). The co-expression of *HaLPCAT1* with *CsFAD2* induced an increase in the proportion of 18:2 and total PUFA content in PC, PE, and TAG. These increases occurred at expense of oleate and the other monounsaturated fatty acids (*n*-7 ones) (Table 2). The effects of expressing either of the other *HaLPCATs* genes were more difficult to interpret. *Ha*LPCAT2 did not significantly alter the distribution of PUFAs in the lipid classes studied whereas *Ha*LPCAT3 significantly increased the 18:1^Δ11^ content of PC. Expression of either of these cDNA resulted in incremental increases in the incorporation of 18:2 into PE, although the impact on the incorporation of PUFAs to the other glycerolipids was not clear. Collectively, the results presented in Tables 1, 2 indicate that the recombinant production of *Ha*LPCATs in *S. cerevisiae* can affect both the proportion of lipid classes and their fatty acid compositions. This result also showed that LPCAT could use other phospholipid species as substrates. If this is confirmed by future research, it could change the view we have about the function of these enzymes in lipid metabolism.

### Influence of Producing Recombinant HaLPCAT on Yeast Acyl-CoA Pools

The acyl-CoA pool of the Y02431 mutant with empty vector contained four components: 16:0-CoA, 16:1-CoA, 18:0-CoA, and 18:1-CoA. 16:0-CoA and 18:1-CoA were the major thioesters observed at levels of 36.1 and 23.4 mole %, respectively. Yeast are not able to desaturate oleate, and thus no 18:2-CoA was detected in the acyl-CoA pool of the control strain (Table 3). The expression of each of the three *HaLPCATs*, however, altered the composition of the acyl-CoA pool. In the absence of *Cs*FAD2, no 18:2-CoA was produced, the changes were the same in all cases, and involved a decrease in the proportion of saturated molecular species of acyl-CoA (16:0 and 18:0) at the expense of monounsaturated species (16:1 plus 18:1) (Table 3). The observed changes were not always significant. When *CsFAD2* was co-expressed, a production of 8.3 mole % 18:2-CoA was observed. This component appeared at expense of significant decreases of 18:1-CoA and 16:1-CoA. Co-expression of each *HaLPCAT* with *CsFAD2* desaturase resulted in further increases in 18:2-CoA. The presence of the LPCATs activates the acyl exchange in PC. A higher turnover of fatty acids avoids the linoleate being stuck in PC blocking desaturation. So, both reactions direct and reverse have a contribution to increase the levels linoleate, the direct esterifying fresh newly synthesized oleate and the reverse removing the linoleate produced by reticular desaturases. These increases were significant for expression of *HaLPCAT1* or *HaLPCAT3*, and a slightly reduced expression for *HaLPCAT2*. This result showed that *Ha*LPCATs are involved in catalyzing acyl-exchange between nitrogenous phospholipids and the acyl-CoA pool as previously shown in studies with developing Arabidopsis seeds (Wang et al., 2014).

**TABLE 3:** Fatty acyl composition of the acyl-CoA pool of *Saccharomyces cerevisiae* strain Y02431 (*slc4*Δ) mutant expressing *LPCATs* from sunflower in the presence or absence of expression of a *Camelina sativa FATTY ACID DESATURASE 2* (*CsFAD2*).

		Composition (mole%)				
		16:0-CoA	16:1-CoA	18:0-CoA	18:1-CoA	18:2-CoA
*CsFAD2*−	p416GPD/p423GPD	36.1 ± 5.2	23.1 ± 2.7	17.4 ± 0.8	23.4 ± 3.5	nd
	p416GPD/p423GPD:*HaLPCAT1*	29.6 ± 6.4	28.8 ± 2.8	16.9 ± 1.6	24.8 ± 5.8	nd
	p416GPD/p423GPD: *HaLPCAT2*	29.4 ± 8.1	27.0 ± 9.6	12.9 ± 2.4*	30.7 ± 3.9*	nd
	p416GPD/p423GPD: *HaLPCAT3*	30.6 ± 10.3	21.9 ± 7.2	15.2 ± 4.8	32.3 ± 9.5*	nd
*CsFAD2*+	p416GPD:CsFAD2/p423GPD	41.5 ± 2.5	20.0 ± 1.0	19.2 ± 2.1	11.0 ± 2.3	8.3 ± 1.9
	p416GPD:CsFAD2/p423GPD: *HaLPCAT1*	40.2 ± 2.0	17.6 ± 3.7	22.1 ± 3.9	6.3 ± 1.6*	13.8 ± 2.2*
	p416GPD:CsFAD2/p423GPD: *HaLPCAT2*	39.8 ± 6.5	15.7 ± 3.7	21.2 ± 2.9	7.3 ± 0.9*	16.0 ± 6.4
	p416GPD:CsFAD2/p423GPD: *HaLPCAT3*	31.9 ± 10.9	17.8 ± 3.5	15.9 ± 6.6	13.9 ± 5.3	20.6 ± 4.2*

*HaLPCATs were expressed in the vector p423GPD, whereas CsFAD2 was expressed in p416GPD. Control lines correspond to empty plasmids. Data are the average of four independent determinations ± standard deviation (*p* > 0.05). Asterisks indicate significant differences with control cells according to one-way ANOVA with Tukey post hoc analysis with a significance level of 0.05%.*

### *In vitro* Assay and Substrate Specificity Studies of HaLPCATs

**FIGURE 6:**
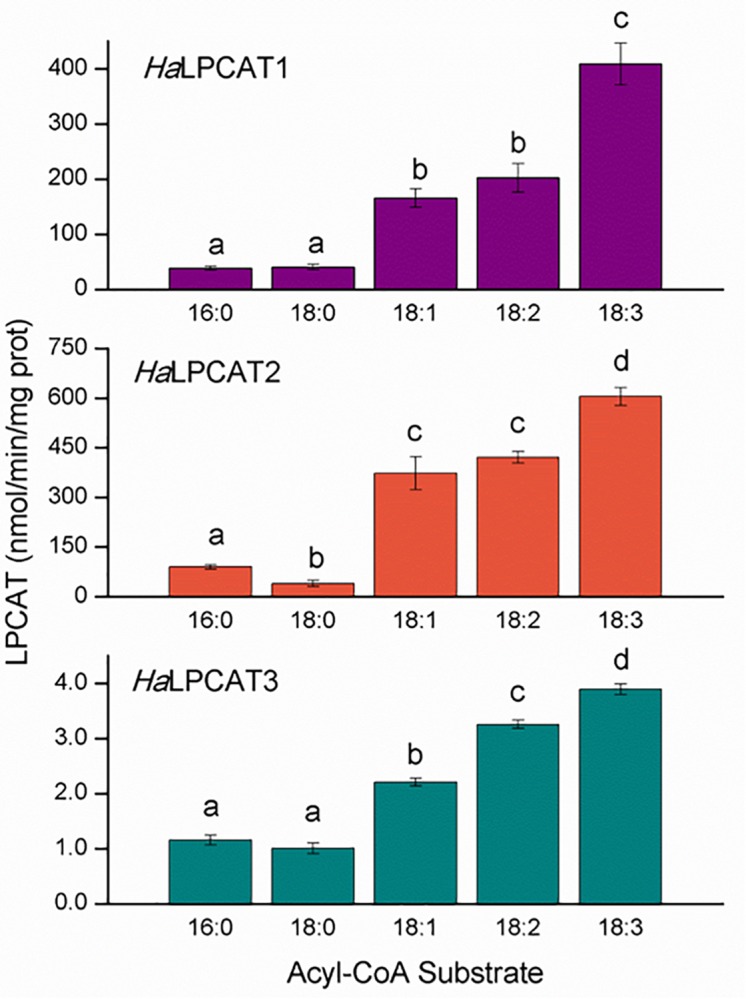
Acyl-CoA specificity of the forward reaction catalyzed by microsomal recombinant HaLPCATs. In all cases, the glycerolipid substrate was *sn*-1-[1-^14^C]oleoyl-lyso phosphatidylcholine. Data are the mean of three replicates with±standard deviation. The letters indicated the groups of statistical significance according to a one-way ANOVA with Tuckey *post hoc* analysis at a significance level of 0.05%.

The enzyme activities of recombinantly produced *Ha*LPCATs were assayed in microsomes isolated from Y02431 yeast strain, which was an ALE1 knockout mutant. This strain did not display any detectable endogenous LPCAT activity (Supplementary Figure S4). Forward reaction activity assays were performed in the range of the first 15 min of reaction and between 1 and 30 μg of microsomal protein. In all cases, measurable activities were found for all substrates assayed (Figure 6), although the specific activity of microsomal *Ha*LPCAT3 was about two orders of magnitude lower than that for *Ha*LPCAT1 or *Ha*LPCAT2. This disparity in absolute activity displayed has been reported in similar studies carried out previously with recombinant LPCATs from other sources (Lager et al., 2013). Nevertheless, the relative substrate specificity for the three enzymes was similar, displaying low activities toward saturated acyl-CoA and higher ones for unsaturated acyl-CoAs (Figure 6). In all three cases, enzyme activity was highest when 18:3-CoA was utilized as an acyl donor. Substrate specificity differences for the forward-catalyzed reaction were classified into four different one-way ANOVA significance groups as shown in Figure 6. In other species, similar results were reported. Thus, Zhang et al. (2015) studied two forms of LPCAT from *N. benthamiana* that displayed the highest direct activity toward 18:3-CoA, followed of 18:2-CoA and 18:1-CoA. The lowest activity was found with 16:0-CoA. In the case of *B. napus*, two forms were also found displaying reactions of acylation of LPCAT that were maximum maximum with 16:1-CoA (Zheng et al., 2012). They displayed similar activities for 18:1-CoA, 18:2-CoA, and 18:3-CoA and lower ones with saturated and very long chain derivatives. Lager et al. (2013) studied the forms of At and other species like *Lesquerella fendleri*, *R. communis*, *Carthamus ticntorius*, *or Hiptage benghalensis* in all cases activities were lower with saturated 16:0-acyl-CoA and higher with unsaturated or polyunsaturated derivatives. A more recent work on *C. sativa* LPCAT measured in microsomes from developing seeds displayed a similar activity profile (Kliñska et al., 2019). This similarity in the acyl-CoA specificity of LPCATs from different species suggests that LPCAT action is central to maintaining the membrane fluidity and functionality. In the light of *HaLPCAT2* or *HaLPCAT3* being the major forms expressed in developing sunflower seeds, it is interesting to note that both isoforms exhibit the highest specificity for 18:3-CoA even though developing sunflower seeds do not accumulate much 18:3 in their TAGs. This result could point a function related with chloroplastic lipids of vegetative tissues, which are specially enriched in 18:3. Thus in the work published by Karki et al. (2019) on the lipid trafficking between the ER and the chloroplast would involve the acylation of LPC by action of LPCAT-catalyzed acyl editing followed by the incorporation of the DAG moiety of a certain population of PC into MGDG. The LPCAT specificity found in this work and other similar to this would support this hypothesis. The reverse-catalyzed reaction of plant LPCAT facilitates enrichment of the acyl-CoA in PUFA (Stymne and Stobart, 1984, 1987). The reverse reaction is less thermodynamically favorable because it requires the production of a high energy thioester (acyl-CoA). Under *in vivo* conditions, acyl-CoA binding proteins might act to reduce local acyl-CoA concentrations so as to encourage the reverse-catalyzed reaction (Stymne and Stobart, 1987; Yurchenko et al., 2009). In addition, the acyl-CoA-dependent acyltransferases of the Kennedy pathway may also participate in favoring the reverse reaction of LPCAT through removal of acyl-CoAs (Pan et al., 2015). The low reverse activity of LPCATs can also be compensated by the enzymes of the Lands cycle, which involves phospholipase A2 (PLA2) hydrolyzing fatty acids from the *sn*-2 position of PC and LACS activity activating those free fatty acids to their acyl-CoAs, which would equilibrate the acyl exchange with the acyl-CoA pool (Wang et al., 2012). This type of PLA2 has an important implication in the metabolism of ricinoleic acid synthetized in PC and accumulated specifically in TAGs by castor seeds (Bayon et al., 2015).

**FIGURE 7:**
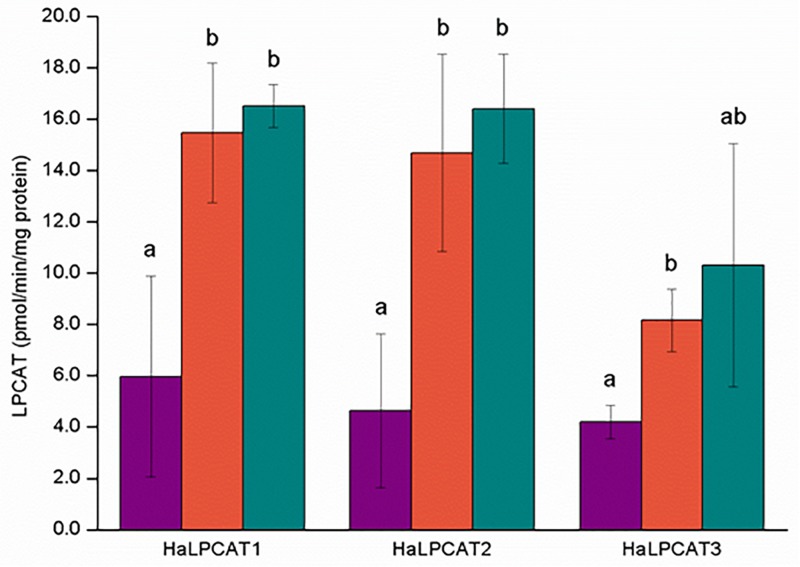
Phosphatidylcholine species specificity of the reverse reaction catalyzed by microsomal recombinant *Ha*LPCATs. The substrates used were *sn*-1,2-dioleoyl-phosphatidylcholine (purple), *sn*-1-oleoyl-*sn*-2-linoleoyl-phosphatidylcholine (orange), and *sn*-1-oleoyl-*sn*-2-linolenoyl-phosphatidylcholine (green). Data are the mean of three replicates with±standard deviation. The letters indicated the groups of statistical significance according to a one-way ANOVA with Tuckey *post hoc* analysis at a significance level of 0.05%.

The specific activities of the reverse-catalyzed reactions of the recombinant microsomal sunflower enzymes ranged from 5 to 18 pmol/min/mg protein and displayed similar substrate specificity in all cases, with activities significantly higher toward PC molecules containing PUFAs esterified in the *sn*-2 position: *sn*-1-18:1-*sn*-2-18:2-PC and *sn*-1-18:1-*sn*-2-18:3-PC (Figure 7). The microsomal specific activities for the reverse-catalyzed reaction were substantially lower than for the forward-catalyzed reactions. Although the activity of *Ha*LPCAT3 was somewhat lower than for the other two isoforms, the difference was not as great as observed for the forward-catalyzed reaction (Figure 6). In general, the reverse-catalyzed reactions for each of the three *Ha*LPCATs displayed similar substrate specificities, with preference for the removal of PUFAs (18:2 or 18:3) from PC with transfer of the acyl moiety to the acyl-CoA pool. These results are in agreement with previous results reported for LPCATs from other species (Lager et al., 2013) and with results reported by Aznar-Moreno et al. (2013) where the acyl-CoA pool of developing sunflower seeds was shown to be enriched in the linoleoyl moieties.

### Studies of Complementation of Atlpcat1/Atlpcat2 Arabidopsis Mutant

The Arabidopsis double mutant *lpcat1* (At1g12640) *lpcat2* (At1g63050), kindly supplied by professor Anders S. Carlsson, from the Swedish University of Agricultural Sciences (Xu et al., 2012), was used for the expression work. This mutant has insertions in the two genes encoding *At*LPCATs and thus hampers their normal expression. Our PCR experiments, however, revealed a residual expression of *AtLPCAT2*, in the approximate range of 2–10% (Supplementary Figure S5). Nonetheless, as shown in Table 4, the mutations within the two *AtLPCATs* resulted in a decrease in the accumulation of seed PUFAs at expenses of an increase of VLCFAs, especially 20:1 (Bates et al., 2012; Table 4). Thus, this mutant displayed significant decreases in 18:3 at expense of 18:1 and 20:1. Each *HaLPCATs* was transferred to the vector pBIN35S for expression in Arabidopsis under the control of the 35S promoter, which induces high levels of expression in most plant tissues and organs (Odell et al., 1985). The expression of each *HaLPCAT* in this mutant Arabidopsis altered the fatty acid composition of the seeds of the host plant. Increases in PUFA content were attributable to increases in the proportions of 18:2 and 18:3 (Table 4). The sunflower LPCATs also resulted in decreases of the VLCFAs, which in the transgenic plants decreased to levels even lower than those in the WT Col-0 plant. This decrease was mainly attributable to molecular species of 20:1. For each *Ha*LPCAT, substantial decreases in seed oil content were also observed. This decrease was similar for the three enzymes was difficult to interpret. Thus, we cannot discard the metabolic stress caused by the overexpression of the LPCAT proteins regulated by the constitutive CaMV 35S promoter could be the cause of the oil decrease. However, the plants did not display any morphological alteration or deficiencies in growth rates. Other possibility could be that sunflower LPCATs altered the acyl flows in At seeds hampering the correct accumulation and assembly of TAGs. A further characterization of the different lipid species of the transformed mutant could give us light on this aspect. Anyhow, the sunflower LPCATs complemented the *Atlpcat1/Atlpcat2* Arabidopsis double mutant, increasing the percentage of PUFAs in its seed oil.

**TABLE 4:** Fatty acid composition of *Arabidopsis thaliana* seeds of the Col-0 line (WT), mutant *Atlpcat1*/*Atlpcat2*, and the mutant transformed with the different *HaLPCAT*s.

	Composition (mol%)																Oil content
	16:0	16:1	18:0	18:1^Δ9^	18:1^Δ11^	18:2	18:3	20:0	20:1^Δ11^	20:1^Δ13^	20:2	20:3	22:0	22:1^Δ13^	PUFAs	VLCFAs	%(w/w)
Col-0	7.1 ± 0.4	0.26 ± 0.03	3.6 ± 0.1	15.2 ± 0.2	1.50 ± 0.03	27.6 ± 0.4	16.7 ± 0.3	2.22 ± 0.08	19.7 ± 0.5	1.7 ± 0.1	1.84 ± 0.06	0.44 ± 0.02	0.36 ± 0.36	1.9 ± 0.13	46.5 ± 0.6	28.2 ± 0.9	24.5 ± 0.4
*Atlpcat1/Atlpcat2*	6.3 ± 0.4	0.26 ± 0.5	3.2 ± 0.1	18.4 ± 1.1*	1.8 ± 0.2	27.3 ± 0.4	12.7 ± 0.5*	1.49 ± 0.06	22.7 ± 0.7*	1.5 ± 0.2	2.1 ± 0.1*	0.34 ± 0.04*	0.31 ± 0.07	1.7 ± 0.2	42.4 ± 0.6*	30.1 ± 1.0	24.0 ± 5.9
*Atlpcat1/Atlpcat2* HaLPCAT1	7.8 ± 0.4	0.47 ± 0.04	3.2 ± 0.1	16.1 ± 1.0^#^	2.1 ± 0.1	29.5 ± 0.5	16.6 ± 0.9^#^	1.28 ± 0.09	17.7 ± 0.7^#^	1.8 ± 0.2	1.9 ± 0.2	0.28 ± 0.05	0.20 ± 0.07	1.1 ± 0.2	48.3 ± 1.1^#^	24.2 ± 1.3*#	16.0 ± 0.6
*Atlpcat1/Atlpcat2* HaLPCAT2	7.9 ± 0.3	0.46 ± 0.03	3.4 ± 0.2	15.8 ± 0.8^#^	2.1 ± 0.2	29.1 ± 0.6	17.2 ± 0.9^#^	1.36 ± 0.08	17.6 ± 0.9^#^	1.8 ± 0.1	1.9 ± 0.1	0.3 ± 0.1	0.28 ± 0.04	1.0 ± 0.1	48.5 ± 1.1^#^	24.2 ± 1.4*#	13.0 ± 1.1
*Atlpcat1/Atlpcat2* Ha*L*PCAT3	8.1 ± 0.3	0.48 ± 0.10	3.4 ± 0.2	13.9 ± 1.1^#^	2.1 ± 0.1	26.8 ± 0.5	17.1 ± 0.8^#^	1.72 ± 0.06	20.0 ± 0.6*#	2.0 ± 0.2	2.3 ± 0.2	0.48 ± 0.07	0.24 ± 0.02	1.4 ± 0.1	46.7 ± 0.1^#^	28.1 ± 1.2	16.6 ± 1.3

*Data are the average of determinations of 50 seeds from 10 independent plants (T3) from three to five homozygous lines ± standard deviation. Asterisks indicate significant differences with wild type (WT) plants and the # sign significant differences with the double mutant according to one-way ANOVA with Tukey *post hoc* analysis with a significance level of 0.05%. Seeds obtained from independent transgenic lines at the T3 generation were screened for expression of the transgenes.*

Collectively, the data in this report indicate that sunflower LPCATs contribute to the routing of 18:2 from PC into the acyl-CoA pool and TAG during seed development.

## Conclusion

In the present work, three LPCAT forms were cloned from developing sunflower seeds. All they were functional and able to change the lipid composition of yeast when used as a host for them. The three LPCATs display different expression pattern and impacted differently in the yeast lipid composition, pointed to different functions in sunflower metabolism. Furthermore, the thee forms contributed to increase the production of polyunsaturated linoleic acid in yeast. Although the forms *Ha*LPCAT1 and *Ha*LPCAT2 were similar to those found in other species, *Ha*LPCAT3 differed in gene expression and functionality. The three enzymes were characterized *in vitro*, displaying specificity profiles which favored the acyl exchange of PUFAs. All the three enzymes complemented the *lpcat1*/*lpcat2* At mutant, recovering the fatty acid composition of the WT plant, although they induced a decrease in the oil accumulated by the seeds. This work opens interesting fields of future research like the activity of LPCATs toward other polar lipids different to PC and their impact on accumulation of TAGs in transgenic At seeds.

## Data Availability Statement

The datasets generated for this study can be found in the GenBank: JN112899, JN112900, and KY235263.

## Author Contributions

EM-F, JS, and MV-C conceived and conducted the project. XP and RW designed and supervised the experiments involving LPCAT assays. AM-B performed most of the experiments, except Arabidopsis mutant confirmation which was performed by RS. AM-P provided technical support for lipid analysis. RG, EM-F, JS, and MV-C analyzed most data. JS and MV-C wrote the manuscript. All authors participated in further editing of the document.

## Conflict of Interest

The authors declare that the research was conducted in the absence of any commercial or financial relationships that could be construed as a potential conflict of interest.

## Footnotes

1 https://www.yeastgenome.org/locus/S000005701

2 http://www.ncbi.nlm.nhi.gov/

3 http://www.sunflowergenome.org/

4 http://www.expasy.org/tools/

5 www.heliagene.com

## Abbreviations

5-FOA: 5 ′ -fluoroorotic acid
CL: cardiolipin
DAF: days after flowering
DAG: *sn*-1,2-diacylglycerol
DGAT: acyl-CoA diacylglycerol acyltransferase
FAD: fatty acid desaturase
G3P: *sn*-glycerol-3-phosphate
GPAT: acyl-CoA *sn-*glycerol-3-phosphate acyltransferase
LPA: lysophosphatidate
LPAAT: acyl-CoA-lysophosphatidate acyltransferase
LPC: lysophosphatidylcholine
LPCAT: lysophosphatidylcholine: acyl-CoA acyltransferase
lyso-PAF: lyso-platelet activating factor
PAP: phosphatidate phosphatase
PC: phosphatidylcholine
PDAT: phospholipid: DAG acyltransferase
PDCT: PC: DAG choline phosphotransferase
PE: phosphatidylethanolamine
PI: phosphatidylinositol
PS: phosphatidylserine
PUFA: polyunsaturated fatty acid
TAG: triacylglycerol
VLCFA: very long chain fatty acid
WT: wild type.
