# Supplementary material for: Functional Characterization of Lysophosphatidylcholine: Acyl-CoA Acyltransferase Genes From Sunflower (Helianthus annuus L.)
Source: Front Plant Sci. 2020 Apr 15;11:403. doi: 10.3389/fpls.2020.00403 (PMC7176023; doi:10.3389/fpls.2020.00403)

**Functional characterization of lysophosphatidylcholine: Acyl-Coa Acyltransferase genes from sunflower (Helianthus annuus L.)**

**Supplementary Material**

**Table S1**. List of oligonucleotides used in this study.

| **GENE/PLASMID** | **OLIGONUCLEOTIDES** | **5´-3´-SEQUENCE** | **UTILISATION** | **RESTRICTION ENZYME SEQUENCE** |
| --- | --- | --- | --- | --- |
| *HaLPCAT*1 | *Bam*HI*HaLPCAT*1F  *Xho*I*HaLPCAT*1R | T**GGATCC**ATGGATATGGAATCAACGGC  GC**CTCGAG**TCACTGTTCTTTTCGGGCTT | pMBL-T,  pYES2 and  p23*GPD* cloning | *Bam*HI  *Xho*I |
|  | q*HaLPCAT*1F  q*HaLPCAT*1R | GCCTCCATGAGACACTAACAT  GAAGAACACATGAAAACGACAGC | qPCR | -  - |
|  | pBINPCAT1F  pBINPCAT1R | TT**GGATCC**ATGGATATGGAATCAACGGC  G**GCTGCAG**TCACTGTTCTTTTCGGGCTT | pBIN19 cloning | *BamH*I  *Pst*I |
| *HaLPCAT*2 | *Bam*HI*HaLPCAT*2F  *Xho*I*HaLPCAT*2R | T**GGATCC**ATGGAAATGGAATCAATGG  CG**CTCGAG**TCACTGCTCTTTCCGGGCTT | pMBL-T,  pYES2 and  p23*GPD* cloning | *Bam*HI  *Xho*I |
|  | q*HaLPCAT*2F  q*HaLPCAT*2R | CCTACACGAAACACTGGCTG  TAAACCTCACTGCTCTTTCCG | qPCR | *-*  *-* |
|  | pBIN*HaLPCAT*2F  pBIN*HaLPCAT*2R | AA**GGATCC**ATGGCGTCGGCGATC  AA**CTGCAG**TCACTGCTCTCTTTCCGGG | pBIN19 cloning | *BamH*I  *Pst*I |
| *HaLPCAT*3 | *Eco*RI*HaLPCAT*3F  *Xho*I*HaLPCAT*3R | GCGGC**GAATTC**ATGAAAATGGAAGAAATGGC  GCG**CTCGAG**TTACTTTACTTTTCGGGCTC | pJET1.2,  pYES2 and  p23*GPD* cloning | *Eco*RI  *Xho*I |
|  | q*HaLPCAT*3F  q*HaLPCAT*3R | AAAGCCGCACACATCCAGAG  CCTGTGGTGGTTCTTGAGAG | qPCR | *-*  *-* |
|  | pBIN*HaLPCAT*3F  pBIN*HaLPCAT*3R | TT**CCCGGG**ATGAAAATGGAAGAAATGGC  GG**GTCGAC**TTACTTTACTTTTCGGGCTCTGG | pBIN19 cloning | *Xma*I  *Sal*I |
| *AtLPCAT*1 | *AtLPCAT*1F  *AtLPCAT*1R | ATGGATATGAGTTCAATGGCTG  TTATTCTTCTTTACGCGGTTTTG | Gene amplification | *-*  *-* |
|  | pBIN*AtLPCAT*1F  pBIN*AtLPCAT*1R | TA**GGTACC**ATGGATATGAGTTCAATGGCTG  G**GTCTAGA**TTATTCTTCTTTACGCGGTTTTG | pBIN19 cloning | *Kpn*I  *Xba*I |
|  | SALK_123480LP ^(1)(2)^  SALK_123480RP ^(1)(2)^  SALK_LBb1 | ACTCGGTTCACAGAACCAGTG  AAGCGCGGGAAACTTACTTAC  GCGTGGACCGCTTGCTGCAACT | lpcat1 lpcat2-2 ^(1) (2)^ | *-*  *-*  *-* |
|  | JW160 ^(1)^  JW217 ^(1)^ | **GCGGCCGC**ATGGATATGAGTTCAATG  **GGCGCGCC**TTATTCTTCTTTACGCGGTTT | lpcat1 lpcat2.2 ^(2)^ | *Not*I  *Asc*I |
|  | RTlpcat1_2for  RTlpcat1_2rev | GCAGGCTCACGGGTTATTTA  TTCTTTACGCGGTTTTGGTC | Detection of 3´ expression | *-*  *-* |
| *AtLPCAT*2 | AtLPCAT2F  AtLPCAT2R | ATGGAATTGCTTGACATGAACTC  TTATTCTTCTTTTCTGGTCTTTGGTC | Gene amplification | *-*  *-* |
|  | pBINAtlpcat2F  pBINAtlpcat2R | TA**GGTACC**ATGGAATTGCTTGACATGAACTC  GG**TCTAGA**TTATTCTTCTTTTCTGGTCTTTGGTC | pBIN19 cloning | *Kpn*I  *Xba*I |
|  | RTlpcat2_2for  RTlpcat2_2rev | GCTGTCTGGCATGGACTGTA  GCGACTAGTGTTTCGTGCAA | Detection of 3´expression | *-*  *-* |
|  | SAIL_1213_G01LP ^(1)^  SAIL_1213_G01RP ^(1)^  SAIL_LB1 | GCTTGAAACACAGCTCGAATC  TAACTTCCCATCAGGCATCAG  GCCTTTTCAGAAATGGATAAATAGCCTTGCTTCC | lpcat1 lpcat2-2 ^(1)^ | *-*  *-*  *-* |
|  | SAIL_357_H01LP ^(2)^  SAIL_357_H01RP ^(2)^  SAIL_LB2 | TTTGGTTACCAATACATGGCG  GAAGGCGACTAGTGTTTCGTG  GCTTCCTATTATATCTTCCCAAATTACCAATACA | lpcat1 lpcat2.2 ^(2)^ | *-*  *-*  *-* |
|  | JW162 ^(1)^  JW218 ^(1)^ | **GCGGCCGC**ATGGAATTGCTTGACATG  **GGCGCG**CCTTATTCTTCTTTTCTGGTCTT | lpcat1 lpcat2-2 ^(1)^ | *Not*I  *Xho*I |
| 18S | 18S-F  18S-R | GGTAGGCGATTGGCTAACATTGTCTGC  GAGACACCAACAGTCTTTCCTCTGCG | Control | -  - |
| Actina | q*HaACT*F  q*HaACT*R | GCTAACAGGGAAAAGATGACTC  ACTGGCATAAAGAGAAAGCACG | Control | *-*  *-* |
|  | *At*ActF  *At*ActR | GGAAGGATCTGTACGGTAAC  TGTGAACGATTCCTGGACCT |  |  |
|  | *At*ActF  *At*ActR | GGAAGGATCTGTACGGTAAC  TGTGAACGATTCCTGGACCT |  |  |
| pMBL-T | M13F  M13R | GTAAAACGACGGCCAGT  AGGAAACAGCTATGACCATG | Construct checking | *-*  *-* |
| pJET1.2 | pJET1.2F  pJET1.2R | CGACTCACTATAGGGAGAGCGGC  AAGAACATCGATTTTCCATGGCAG | Construct checking | *-*  *-* |
| pYES2 | GAL1F  CYCR | GTCAAGGAGAAAAAACCCCGGATC  GCGTGACATAACTAATTACATGATGCG | Construct checking | *-*  *-* |
| p423*GPD* and  p416*GPD* | GPDF  CYC2R | AAGTATATAAAGACGGTAGG  ATAGGGACCTAGACTTCAGG | Construct checking | *-*  *-* |
| pBIN19::35S | pBIN19F  pBIN19R | GAAAGGGGGATGTGCTGCAAGG  CACACAGGAAACAGCTATGACC | Construct checking | *-*  *-* |

^(1)^ Wang et al., 2012 ^(2)^ Xu et al., 2012

| **Table S2**. Efficiency of amplification and correlation coefficient to the model of Livak and Schmittgen (2001) for the 3 LPCAT genes from sunflower and the control actin gene. | | |
| --- | --- | --- |
| Gene | Efficiency | R2 |
|  | (%) |  |
| HaLPCAT1 | 92.4 | 0.997 |
| HaLPCAT2 | 102.6 | 0.984 |
| HaLPCAT3 | 105.1 | 0.997 |
| HaActin | 100.0 | 0.996 |
|  |  |  |

**Figure S1.** Sequence alignment of HaLPCATs with the related LPCATs from higher plants with their different motifs and the RE signal. Identical residues are highlighted as black boxes, highly conserved residues as dark grey boxes and semiconservative changes as light grey boxes.


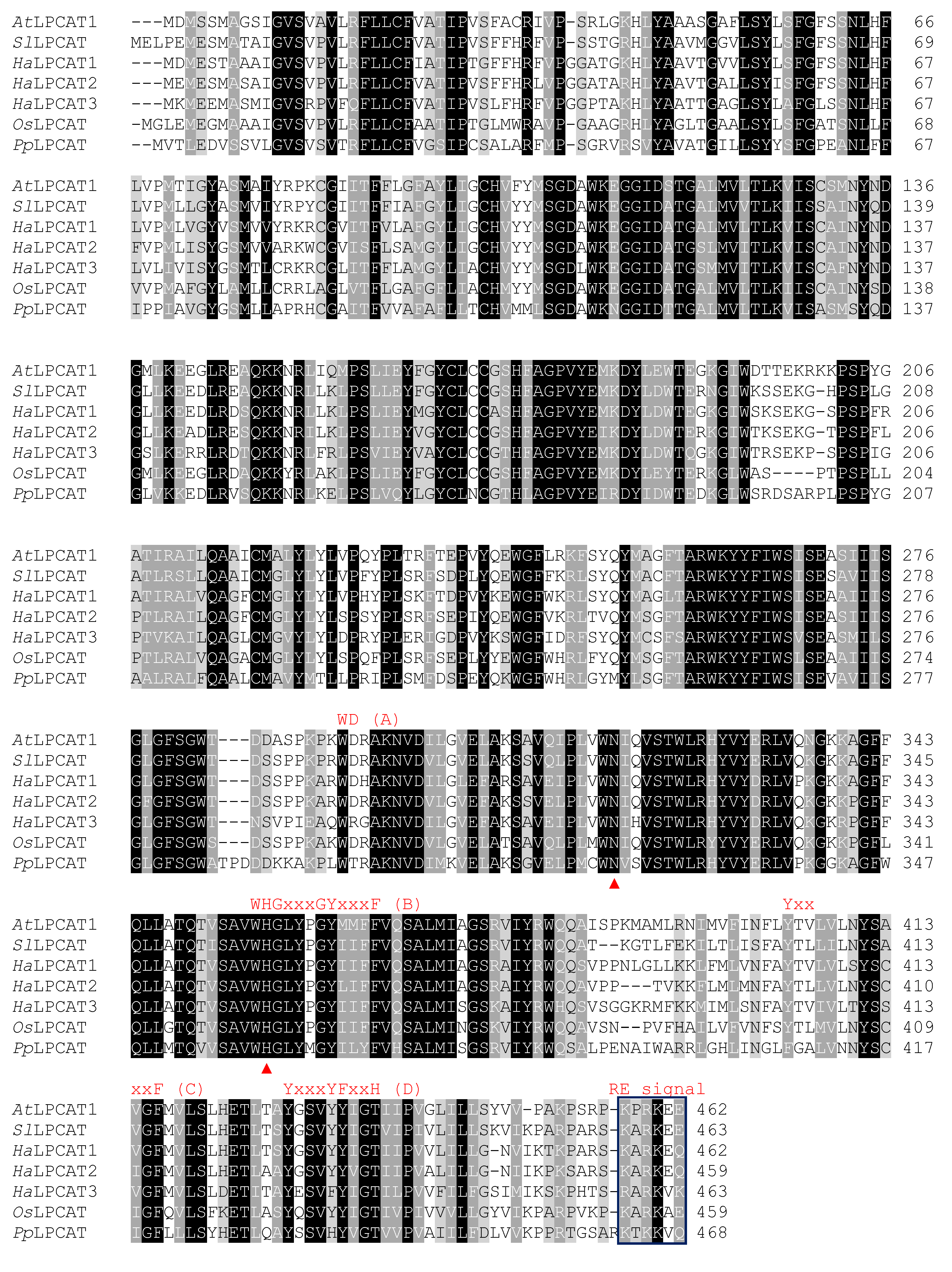


**Figure S2.** Hydrophobocity graphs of TMDs of HaLPCATs.

**HaLPCAT 1**


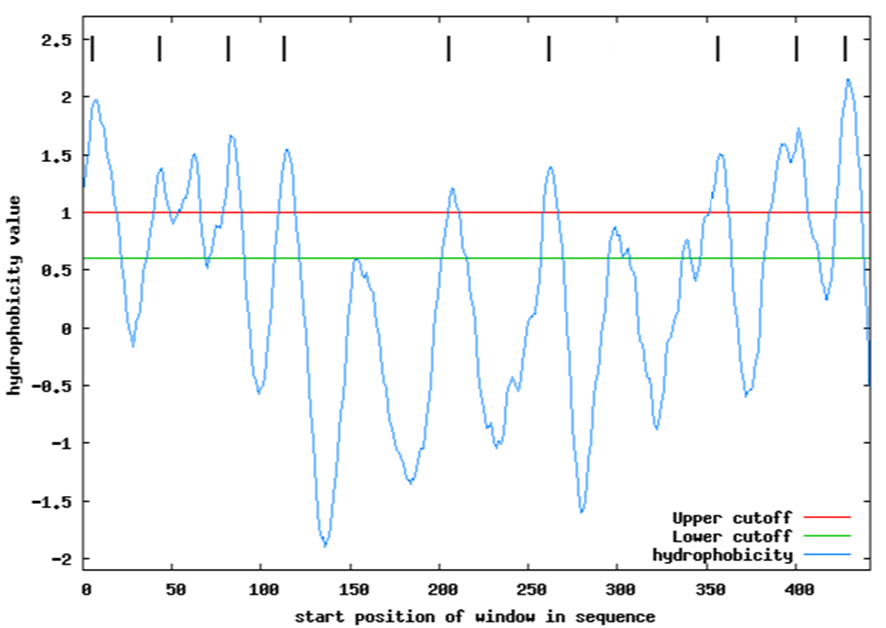


**HaLPCAT 2**


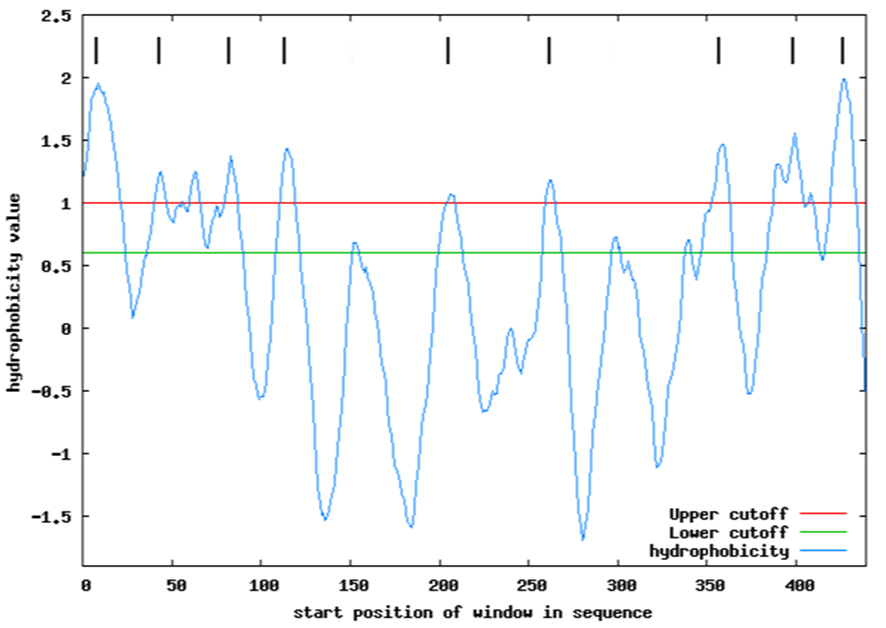


**HaLPCAT 3**


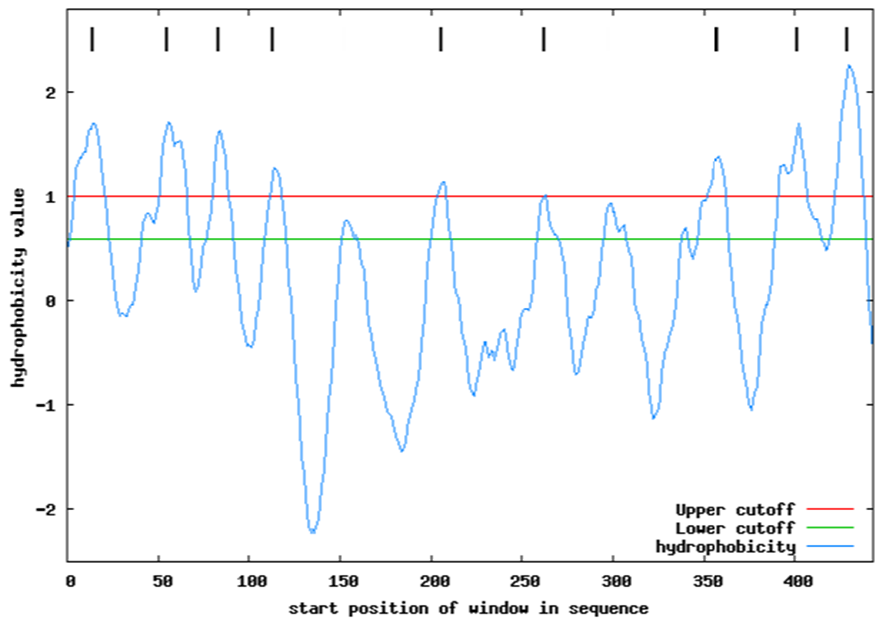


**Figure S3.** Subcellular location prediction of HaLPCAT proteins.


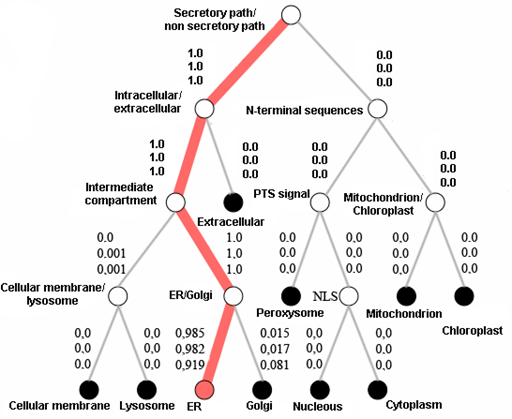


Figure S4. Studies on the influence of reaction time (A) and amount of protein (B) in the activity HaLPCAT1 in the direct reaction assayed in yeast microsomes. Data represent the average value of three independent determinations plus minus standard deviation. pYES2::*HaLPCAT*1 (-■-), empty pYES2 (-●-).


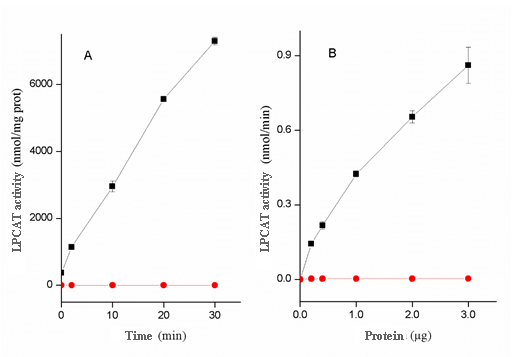


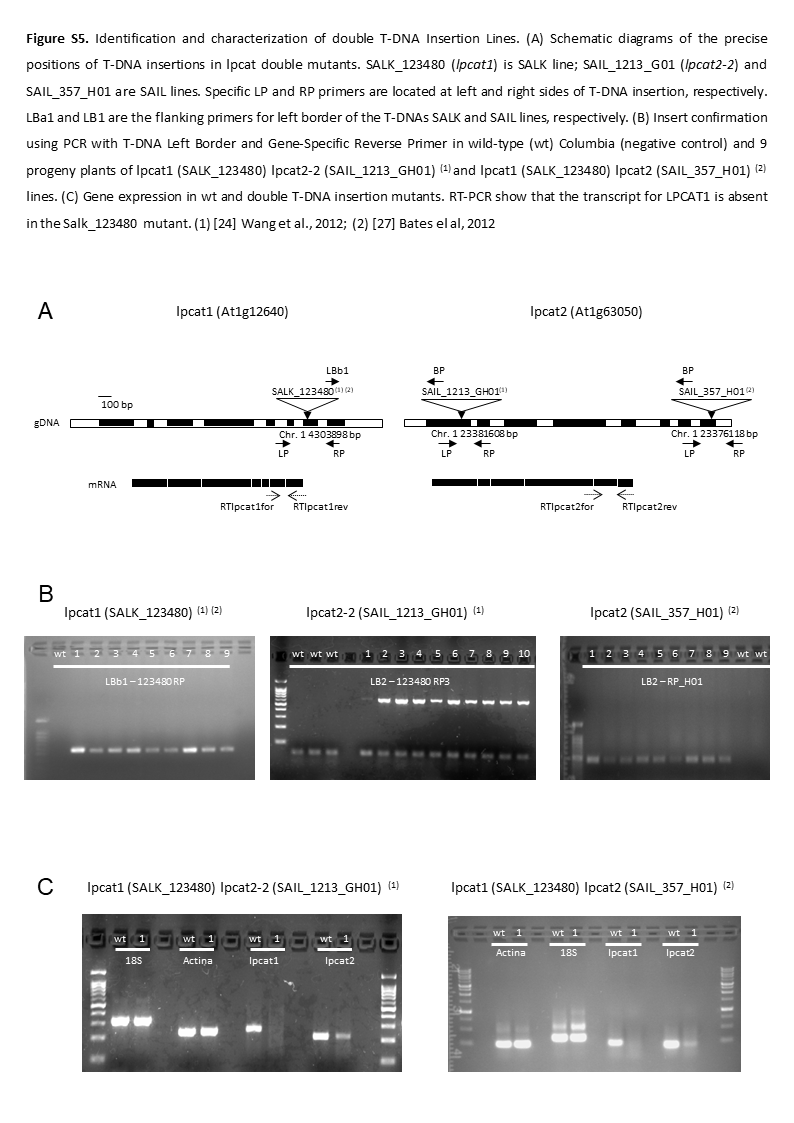

Supplement: Supplementary file 1 [file Data_Sheet_1.docx]
